# Profiling the Production of Antimicrobial Secondary Metabolites by *Xenorhabdus khoisanae* J194 Under Different Culturing Conditions

**DOI:** 10.3389/fchem.2021.626653

**Published:** 2021-03-30

**Authors:** Elzaan Booysen, Marina Rautenbach, Marietjie A. Stander, Leon M. T. Dicks

**Affiliations:** ^1^ Department of Microbiology, Stellenbosch University, Stellenbosch, South Africa; ^2^ Department of Biochemistry, Stellenbosch University, Stellenbosch, South Africa; ^3^ LCMS Central Analytical Facility, Stellenbosch University, Stellenbosch, South Africa

**Keywords:** *Xenorhabdus khoisanae*, secondary metabolites, antimicrobial peptides, UPLC- MS, culture conditions

## Abstract

Species from the genus *Xenorhabdus,* endosymbiotic bacteria of *Steinernema* nematodes, produce several antibacterial and antifungal compounds, some of which are anti-parasitic. In this study, we report on the effect growth conditions have on the production of antimicrobial compounds produced by *Xenorhabdus khoisanae* J194. The strain was cultured in aerated and non-aerated broth, respectively, and on solid media. Production of antimicrobial compounds was detected after 24 h of growth in liquid media, with highest levels recorded after 96 h. Highest antimicrobial activity was obtained from cells cultured on solid media. By using ultraperformance liquid chromatography linked to mass spectrometry and HPLC, a plethora of known *Xenorhabdus* compounds were identified. These compounds are the PAX lipopeptides (PAX 1′, PAX 3′, PAX 5, and PAX 7E), xenocoumacins and xenoamicins. Differences observed in the MS-MS fractionation patterns collected in this study, when compared to previous studies indicated that this strain produces novel xenoamicins. Three novel antimicrobial compounds, khoicin, xenopep and rhabdin, were identified and structurally characterized based on MS-MS fractionation patterns, amino acid analysis and whole genome analysis. The various compounds produced under the three different conditions indicates that the secondary metabolism of *X. khoisanae* J194 may be regulated by oxygen, water activity or both. Based on these findings *X. khoisanae* J194 produce a variety of antimicrobial compounds that may have application in disease control.

## Introduction

Careless and frequent use of antibiotics have led to an increase in resistance (D’Costa et al., 2011; Fair and Tor, 2014; Ventola, 2015). With only a handful novel classes of antibiotics approved during the last three decades (Spellberg et al., 2008), many researchers are of the opinion that we have entered a post-antibiotic era (Lee Ventola, 2015). Novel antimicrobial compounds with modes of activity different to traditional antibiotics may be the answer. This is, however, a challenge, as many newly discovered antimicrobial compounds do not pass stringent safety tests, or production costs are too high.

Approval of daptomycin, an antimicrobial lipopeptide from *Streptomyces roseosporus*, by the Food and Drug Administration (FDA) in 2003 (Arbeit et al., 2004) and the re-approval of polymyxin B, produced by *Bacillus polymyxa* (Zavascki et al., 2007), led to renewed interest in antimicrobial peptides. Most bacteria produce antimicrobial compounds, either for survival, or as a defense mechanism. Many of these compounds target cellular structures such as cell walls and membranes, pathways involved in energy (ATP)-production, or protein- and, nucleic acid synthesis (Kaufman, 2011).

Bacteria living in symbiosis with soil dwelling nematodes produce several antimicrobial compounds. *Xenorhabdus*, a member of the family *Enterobacteriaceae*, is closely associated with *Steinernema* entomopathogenic nematodes that infect the larvae of *Galleria mellonella* Linnaeus (Dunphy and Webster, 1991). A single, or at the most only a few strains, of a *Xenorhabdus* sp. develops a symbiotic relationship with the nematode and keeps the host free from other bacteria. This suggests that the invading strain(s) produce either a single broad spectrum antimicrobial compound, or several compounds. As soon as the nematode invades the insect, *Xenorhabdus* elicits the production of a plethora of secondary metabolites, including antimicrobial peptides, polyketides, proteases and hydrolytic exo-enzymes (Dreyer et al., 2018). In the case of *Xenorhabdus budapestensis*, production of the immune suppressor prophenoloxidase leads to an increase in quinones, killing the insect host (Yang et al., 2012).

A total of 23 antimicrobial compounds produced by *Xenorhabdus* spp. have been described, of which the majority have bactericidal and fungicidal activity (reviewed by Dreyer et al., 2019). These include xenocoumacins (Park et al., 2009; Reimer et al., 2009), bicornitun (Böszörményi et al., 2009), PAX peptides (Gualtieri et al., 2009; Dreyer et al., 2019), xenorhabdins (McInerney et al., 1991a; McInerney et al., 1991b), xenorxides (Webster et al., 1996) and benzylideneacetone (Ji et al., 2004). A few antimicrobial compounds are also insecticidal and anti-parasitic (Ji et al., 2004; Crawford et al., 2011; Crawford et al., 2012; Grundmann et al., 2013; Zhou et al., 2013; Fuchs et al., 2014; Kronenwerth et al., 2014; Proschak et al., 2014; Reimer et al., 2014; Zhao et al., 2018; Hacker et al., 2018). Also refer to review by Booysen and Dicks (2020) that focusses on the potential of *Xenorhabdus* for antibiotic production.

Xenocoumacins are one of the major antimicrobial compounds produced by *Xenorhabdus* spp. and was first described by McInerney et al. (1991a, b). These compounds are known as benzopyran-1-one derivates encoded by the *xcnA-N* gene cassette on the genome of *Xenorhabdus nematophilia* (Park et al., 2009)*.* A total of six xenocoumacins were identified, although xenocoumacins I and II are the two mainly expressed by the *xcnA-N* gene cassette (Reimer et al., 2009). Both xenocoumacins I and II show antimicrobial activity, although xenocoumacin I is more active and kills bacteria and fungi (McInerney et al., 1991b).

Bicornitun is an arginine-rich non-ribosomal antifungal peptide produced by *X. budapestensis,* first described by Böszörményi et al. (2009). Four different bicornitun compounds have been described, i.e., bicornitun A1, A2, B and C. Lysine-rich cyclic lipopeptides, the PAX peptides, were first reported by Gualtieri et al. (2009). An additional eight PAX peptides were later described by Fuchs et al. (2011). Genes encoding the PAX peptides are located in the *paxABC* gene cassette (Fuchs et al., 2011). PAX 5 is active against *Pseudomonas aeruginosa* and *Escherichia coli,* while PAX 3, PAX 4 and PAX 5 are active against *Streptococcus epidermidis.* All PAX peptides are active against *Fusarium oxysprum.*(Gualtieri et al., 2009) No cytotoxic activity was recorded against Chinese Hamster Ovary cells (Gualtieri et al., 2009).

Xenorhabdins and xenorxides are dithiolopyrrolones derivatives, first described by McInerney et al. (1991a, b) and Webster et al. (1996), respectively. These compounds are known for their wide-spread antibacterial, antifungal and insecticidal activity (McInerney et al., 1991a; McInerney et al., 1991b; Li et al., 2014). Benzylideneacetone, produced by *X. nematophilia,* is used as a flavoring additive in cigarettes, food products, detergents and cosmetics (Dreyer et al., 2018). Although this compound have been used in industry for decades, the antibacterial activity of benzylideneacetone was only discovered in 2004 (Ji et al., 2004).

Most of the *Xenorhabdus* antimicrobial compounds are produced by non-ribosomal peptide synthesis (NRPS) (Fuchs et al., 2011; Zhou et al., 2013; Kronenwerth et al., 2014; Hacker et al., 2018), or a combination of polyketide synthesis (PKS) and NRPS (NRPS/PKS) (Qin et al., 2013; Fuchs et al., 2014; Guo et al., 2017). Although not that common, some antimicrobial peptides are also produced by ribosomal peptide synthesis (RPS) (Singh and Banerjee, 2008). Examples of antimicrobial compounds produced by NRPS are xenoamicins (Zhou et al., 2013), PAX peptides (Fuchs et al., 2011) and nematophin (Cai et al., 2017), while xenocoumacins and fabclavins are produced by NRPS/PK synthesis (Guo et al., 2017). Xenocin (Singh and Banerjee, 2008) and xenorhabdicin (Thaler et al., 1995) are the only two RPS peptides described to date. For a more extensive review on antimicrobial compound synthesis by *Xenorhabdus* spp., the reader is referred to Booysen and Dicks (2020). *Xenorhabdus* peptides are usually synthesized by the non-ribosomal synthetases, hybrid non-ribosomal/polyketide synthetases or ribosomes and can include fatty acid or polyketide chains (Gualtieri et al., 2009; Reimer et al., 2011; Zhou et al., 2013), while non-peptide compounds like benyzlideacetone does not contain any amino groups (Dreyer et al., 2018).

Only a few reports have been published on growth conditions affecting the production of antimicrobial compounds, and only those produced by *X. nematophilia* and *Xenorhabdus bovienii*. These included the effect of pH (Wang et al., 2011b), incubation temperature (Chen et al., 1996; Wang et al., 2008a), agitation, aeration (Wang et al., 2008a) and nutrients(Chen et al., 1996; Wang et al., 2008b; Crawford et al., 2010; Wang et al., 2011a). In this study, we report on secondary metabolites and antimicrobial compounds produced by *X. khoisanae* J194, a bacterium indigenous to South Africa identified by Ferreira et al. (2013), and the production of these compounds when cells were cultured in aerated broth, non-aerated broth, and on the surface of solid media.

## Results

### Influence of Culturing and Extract Procedure on Antimicrobial Activity

**FIGURE 1:**
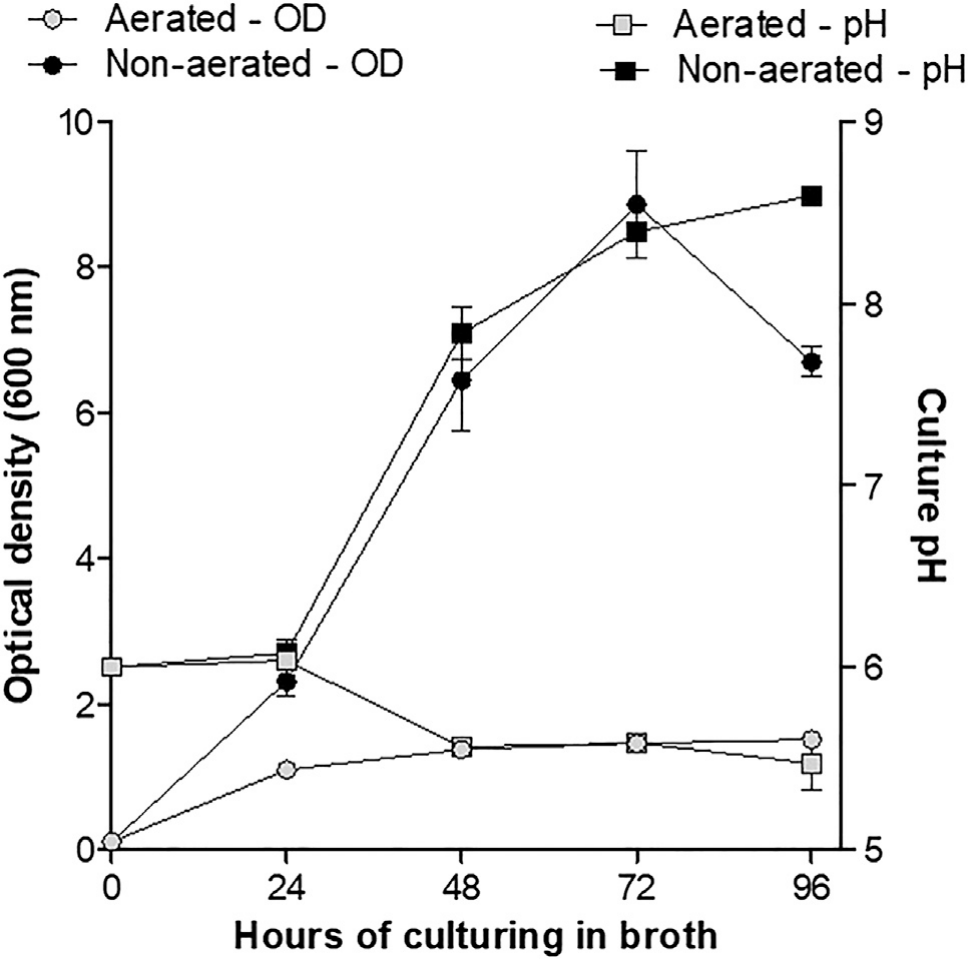
Changes in growth (optical density) and culture pH of *X. khoisanae* J194 under non-aerated (method A) and aerated (method B) culturing in broth over 4 days. Data depicts the mean of the three independent cultures. Error bars show standard deviation.

Various studies have shown that culturing conditions have an effect on bacterial metabolism and production of secondary metabolites. This study showed that the pH of non-aerated cultures (method A) increased from 6.0 to 8.5 over 96 h, while the pH of cells grown under aerated conditions (method B) decreased from 6.0 to 5.5 over the same period (Figure 1). Compared to the aerated cultures (method B) a rapid increase in OD (biomass) was observed for the non-aerated cultures (method A) of strain J194 (Figure 1). Cells of J194 cultured under oxygen-limiting conditions always produced a yellow pigment, that was not observed to the same extent when J194 was cultured under oxygen rich conditions.

During 96 h of growth under non-aerated conditions, 4,196 mg freeze-dried material was collected from CFSs. Of this, 2,863 mg was from the oily bottom fraction (NAO, oily fraction from a non-aerated broth culture) and the rest from the acetonitrile upper fraction (NAA, acetonitrile fraction of a non-aerated broth culture). Over the same period, 1,010 mg freeze-dried material was isolated from the CFSs of aerated cultures (AB, fraction from an aerated culture). Cells grown on the surface of TSA plates produced 5,504 mg of hydrophobic/amphipathic material (SM, extract from a culture grown on the surface of solid media). Less than 100 mg of intracellular material was extracted from aerated and non-aerated cultures.

**FIGURE 2:**
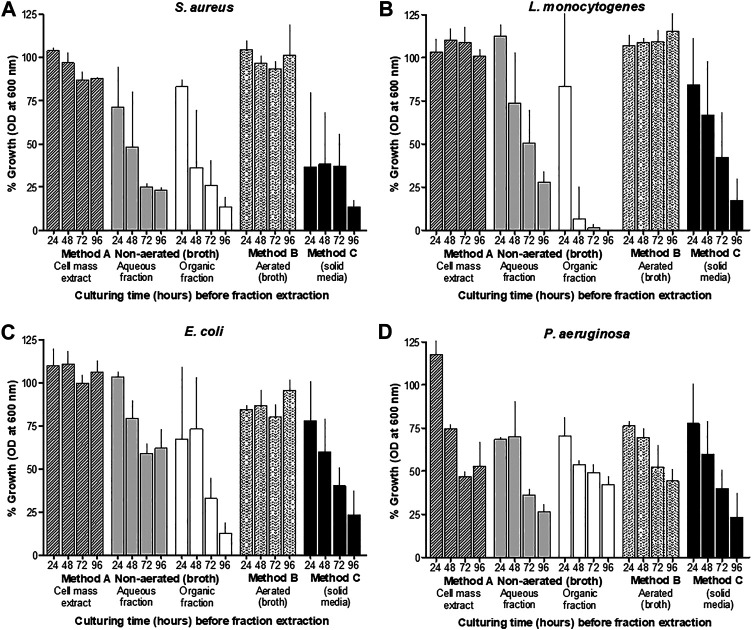
Growth inhibition (%) of different target bacteria*, S. aureus ***(A)***, L. monocytogenes ***(B)***, E. coli ***(C)*** and P. aeruginosa ***(D)***,* as observed by changes in OD at 600 ± 5 nm, compared to control cultures, after exposure to the five different *X. khoisanae* J194 culture extracts. The response (OD at 600 nm) of the selected target bacteria *S. aureus* ATCC 25923, *L. monocytogenes* EDGe, *E. coli* Xen 14 and *P. aeruginosa* ATCC 27853, are shown after 24 h exposure to extracts and incubation at 37°C. Three cultures of each target organism were treated with cell-free supernatants (CFS) of *X. khoisanae* J194 cultured in non-aerated broth (Method A), aerated broth (Method B) and on the surface of solid media (Method C), respectively. CFSs of non-aerated culture were separated into the acetonitrile (NAA) and organic fractions (NAO) (Method A). The hours indicated in the graphs refer to the time at which CFSs were collected and freeze-dried.

The antimicrobial activity of material collected from the CFSs of non-aerated and aerated cultures, and from surface-grown cells after treatment with XAD beads, varied considerably. After 24 h of incubation, antimicrobial activity was recorded in CFSs collected from non-aerated cultures and cultures grown on the surface of TSA (Figure 2). However, after 48 h of incubation in liquid media, all samples, except for the biomass extract, inhibited the growth of *Staphylococcus aureus* ATCC 25923, *Listeria monocytogenes* EDGe, *Escherichia coli* Xen 14 and *Pseudomonas aeruginosa* ATCC 27853. Highest levels of activity were recorded after 96 h of incubation in non-aerated broth and on the agar surface (Figure 2). NAA material collected using method A showed highest activity against *E. coli* Xen 14, while AB material collected using method B and hydrophobic material collected using method C showed the highest activity against *P. aeruginosa* ATCC 27853 (Table 1). Overall, hydrophobic compounds isolated with method C (SM fractions) displayed the highest specific activity against *P. aeruginosa* ATCC 27853 (350 mm^2^/mg)*, E. coli* Xen 14 (263 mm^2^/mg), *S. aureus* Xen 31 (275 mm^2^/mg) and *L. monocytogenes* EDGe (263 mm^2^/mg) (Table 1). NAA material collected from of non-aerated cultures were not active against *S. aureus* ATCC 25923, *S. aureus* Xen 31 and *Streptococcus epidermidis* SE1 (Table 1)*.*

**TABLE 1:** Antibacterial activity of extracts collected from *Xenorhabdus* cultures grown under different conditions.

Bacterial target	Non-aerated, acetonitrile phase extract (NAA)	Non-aerated, oily phase extract (NAO)	Aerated broth extract (AB)	Solid media extract (SM)
*P. aeruginosa* ATCC 27853	100	175	300	350
*E. coli* Xen 14	175	150	150	263
*S. aureus* ATCC 25923	0	100	163	113
*S. aureus* Xen 31	0	138	250	275
*S. epidermidis* SE1	0	163	175	150
*L. monocytogenes* EDGe	63	113	213	263

Activity is indicated by clear zone area (mm^2^/mg) in the agar well diffusion assay.

### UPLC-MS Fingerprinting of Culture Extracts

**FIGURE 3:**
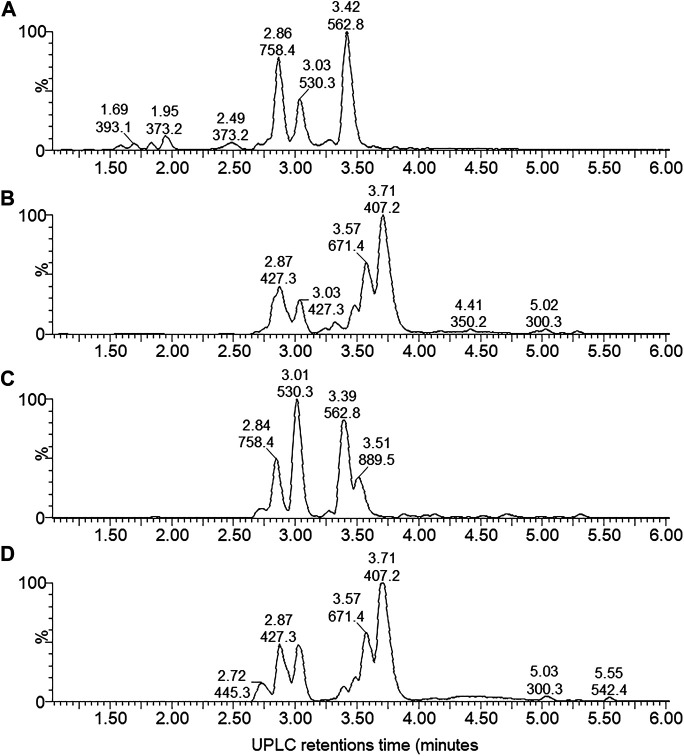
UPLC-MS of the early eluting components (0–6 min) from the four *X. khoisanae* J194 extracts that showed antibacterial activity: **(A)** aqueous fraction of non-aerated broth culture, **(B)** NAO fraction of non-aerated broth culture, **(C)** fraction from aerated broth culture and **(D)** extract from culture grown on surface of solid medium. The masses in the spectra are protonated masses of the most prominent detected compounds.

**FIGURE 4:**
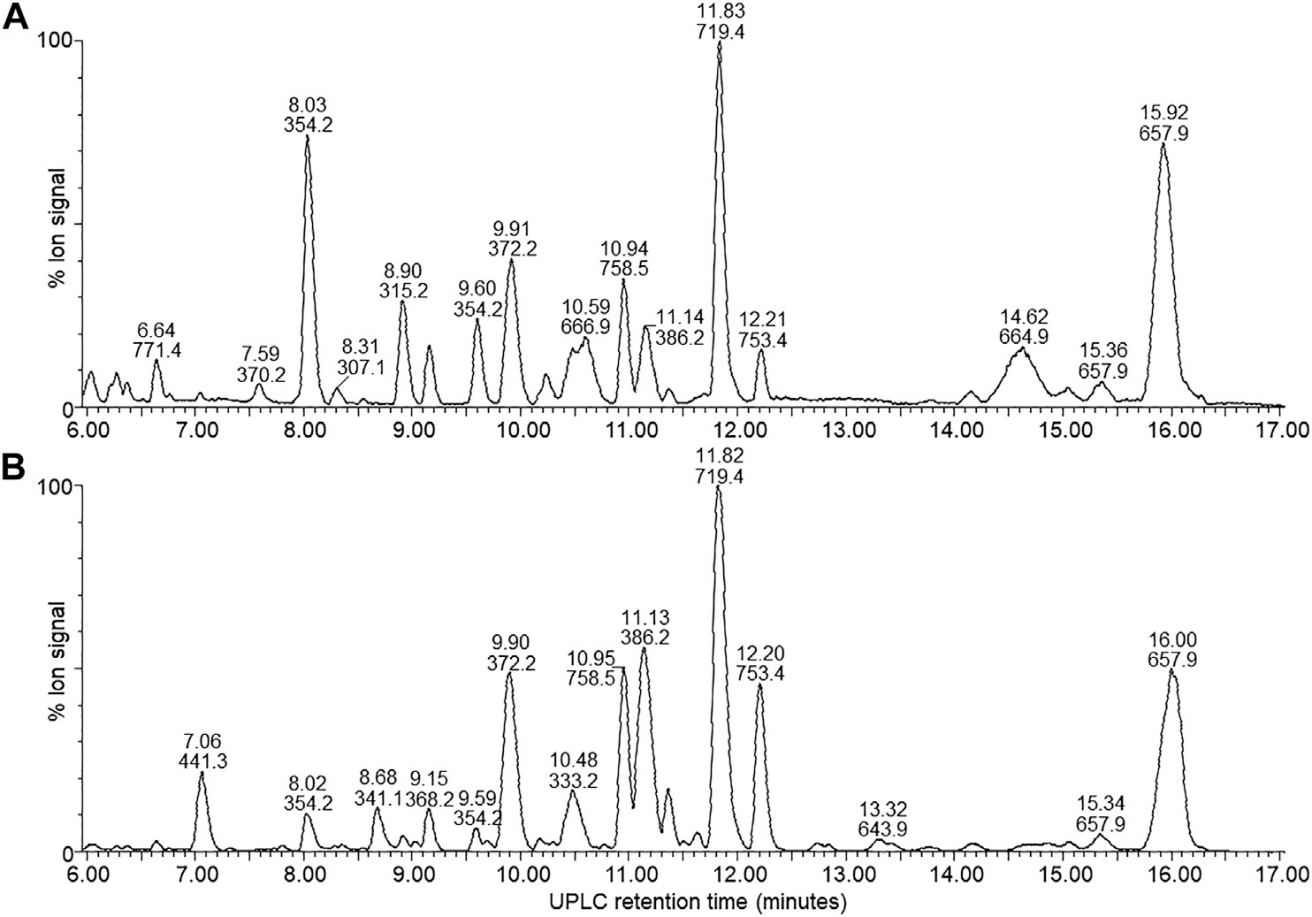
UPLC-MS of the mid-gradient eluting components (6–17 min) from the four X. khoisanae J194 extracts that showed antibacterial activity: **(A)** NAO fraction of nonaerated culture in broth, **(B)** extract from culture grown on surface of solid media. The masses in the spectra are protonated masses of the most prominent detected compounds.

By performing UPLC-MS, we characterized the compounds from the antibiotic/antimicrobial complex based on elution from the C_18_ matrix, signal intensity and accurate Mr. The compounds were grouped into early eluting compounds (0–6 min), representing polar/hydrophilic and amphipathic compounds (Figure 3) and the mid-to-late gradient eluting compounds (6–17 min), representing amphipathic and hydrophobic compounds (Figure 4).

A complex mixture of 44 novel metabolites were selected on the grounds of ion intensity and confirmation of an elution peak in the UPLC-MS analysis (Figures 3, 4; Supplementary Figures S1–5 and Table 2). From these analyses we generated a heatmap (Table 2) to fingerprint and compare the culture conditions and extract fraction. From the fingerprinting we observed that the more polar and amphipathic compounds (Rt 0–6 min) were found in the broth culture extracts (NAA, NAO and AB) (Figure 3; Table 2). The more amphipathic and hydrophobic compounds eluting from 7–16 min were observed primarily in the solid media extract (SM) with some in the oily fraction of the non-aerated broth culture extract (NAO) (Figure 4; Table 2)

**TABLE 2:** Heatmap of UPLC-MS fingerprinting of secondary metabolite profiles in the extracts of the three different *Xenorhabdus* cultures (refer to Figures 3, 4).

Compound Number	Rt (min)	Culture extract				Monoisotopic *M*_r_ of major cations (1+) in UPLC run
		NAA	NAO	AB	SM	
1	2.74	1	1	98	0	479.298
2	2.75	49	16	32	4	652.401
3	2.77	4	30	8	59	445.313
4	2.81	37	24	38	2	751.367
5	2.84	45	21	32	2	758.407
6	2.85	29	34	30	7	558.330
7	2.89	17	44	27	11	627.349
8	2.92	8	53	12	26	**427.302**
9	2.98	1	0	99	0	474.342
10	2.98	17	25	36	23	473.275
11	3.01	33	24	32	12	1100.576
12	3.02	30	9	59	3	530.297
13	3.02	54	27	18	1	1025.567
14	3.16	91	7	2	0	1078.806*
15	3.40	48	4	43	4	**1124.634**
16	3.50	9	29	49	13	**889.481**
17	3.59	5	60	7	28	**671.413**
18	3.75	2	68	0	29	407.217*
19	4.42	4	39	7	50	434.265
20	7.05	3	2	10	86	881.543
21	8.02, 9.59	3	46	1	50	354.220
22	8.93	4	12	5	80	758.455
23	9.90	3	28	4	66	394.211
24	9.90	1	14	2	83	372.229
25	10.47	5	28	4	62	355.198
26	10.49	1	18	1	80	333.217
27	11.14	1	7	2	90	386.244
28	11.35	2	5	4	89	792.416
29	11.82	1	17	4	77	719.432
30	11.83	2	1	11	87	1437.882
31	11.85	5	28	12	56	741.431
32	11.90	3	13	2	83	347.235
33	12.20	2	7	2	89	753.433
34	13.33	0	7	9	84	1286.809
35	13.7, 14.2, 14.7	0	24	0	76	1322.803
36	13.8, 14.2, 14.8	0	15	1	84	1300.823
37	14.63	0	0	4	96	1330.840
38	14.74	0	78	0	22	1350.832
39	14.86	0	66	2	32	1328.853
40	14.89	0	7	1	92	1312.822
41	15.97	0	16	1	83	1331.860
42	15.97	0	17	1	82	**1314.838**
43	15.97	0	23	7	70	1352.794
44	15.99	0	32	1	67	**1336.817**

The ions were selected from the top 50 most abundant ions after confirmation in the UPLC-MS profile. Refer to Supplementary data on analyses.

**FIGURE 5:**
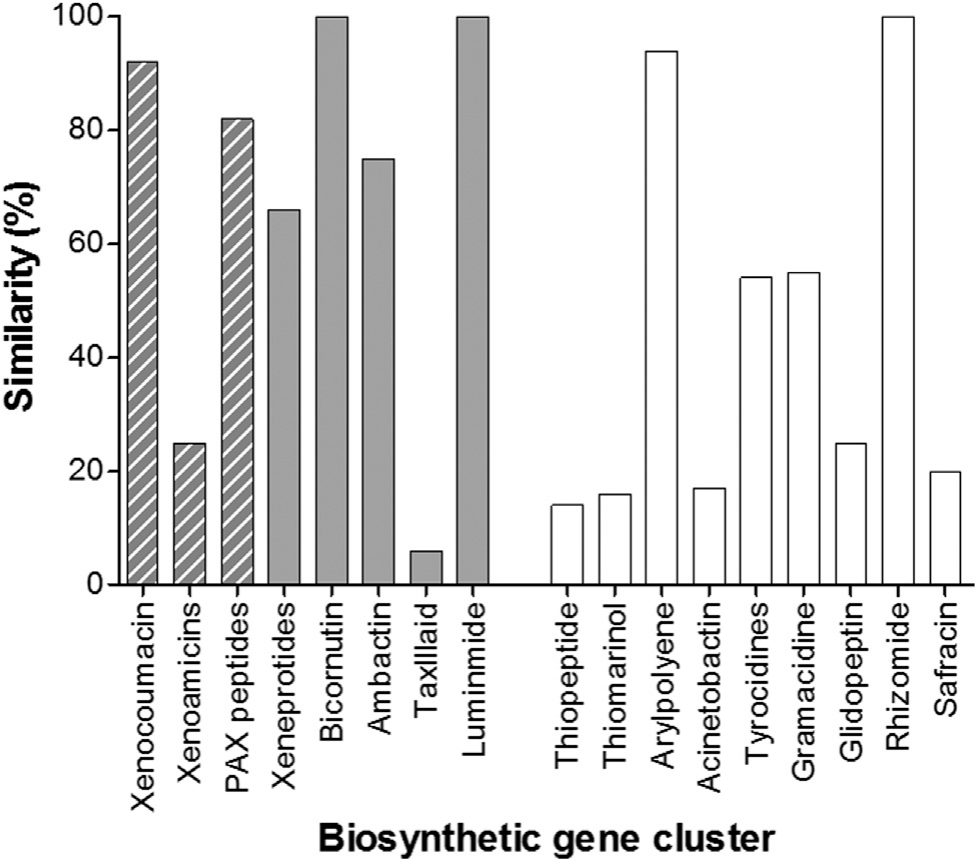
Graph depicting the various operons identified in the whole genome of *X. khoisanae* J194 by the antiSMASH algorithms. The gray bars indicate operons native to *Xenorhabdus* bacteria. Operons for xeneprotides, bicornutin, ambactin, taxlllaid and luminmide were also detected by antiSMASH, but was not identified in the crude extracts. The gray hashed bars indicate detected secondary metabolite products related to the expression of these genes (refer to Table 3). The clear bars indicate other putative operons identified in the whole genome.

**FIGURE 6:**
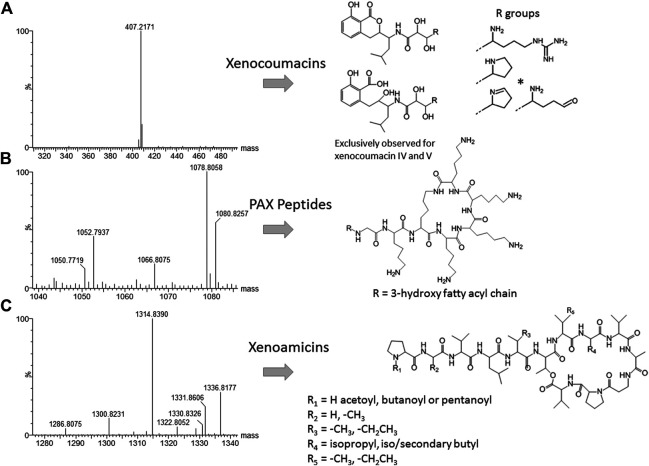
High resolution MS analysis and the structures of compounds in the three most prominent known families identified in J194 culture: **(A)** Xenocoumacin II, identified with a * (McInerney et al., 1991b; Reimer et al., 2009); **(B)** PAX peptides (Fuchs et al., 2011; Dreyer et al., 2019), and **(C)** xenoamicins (Zhou et al., 2013 and this study). Refer to Supplementary Figure S6, S7 for more structural analyses details.

**FIGURE 7:**
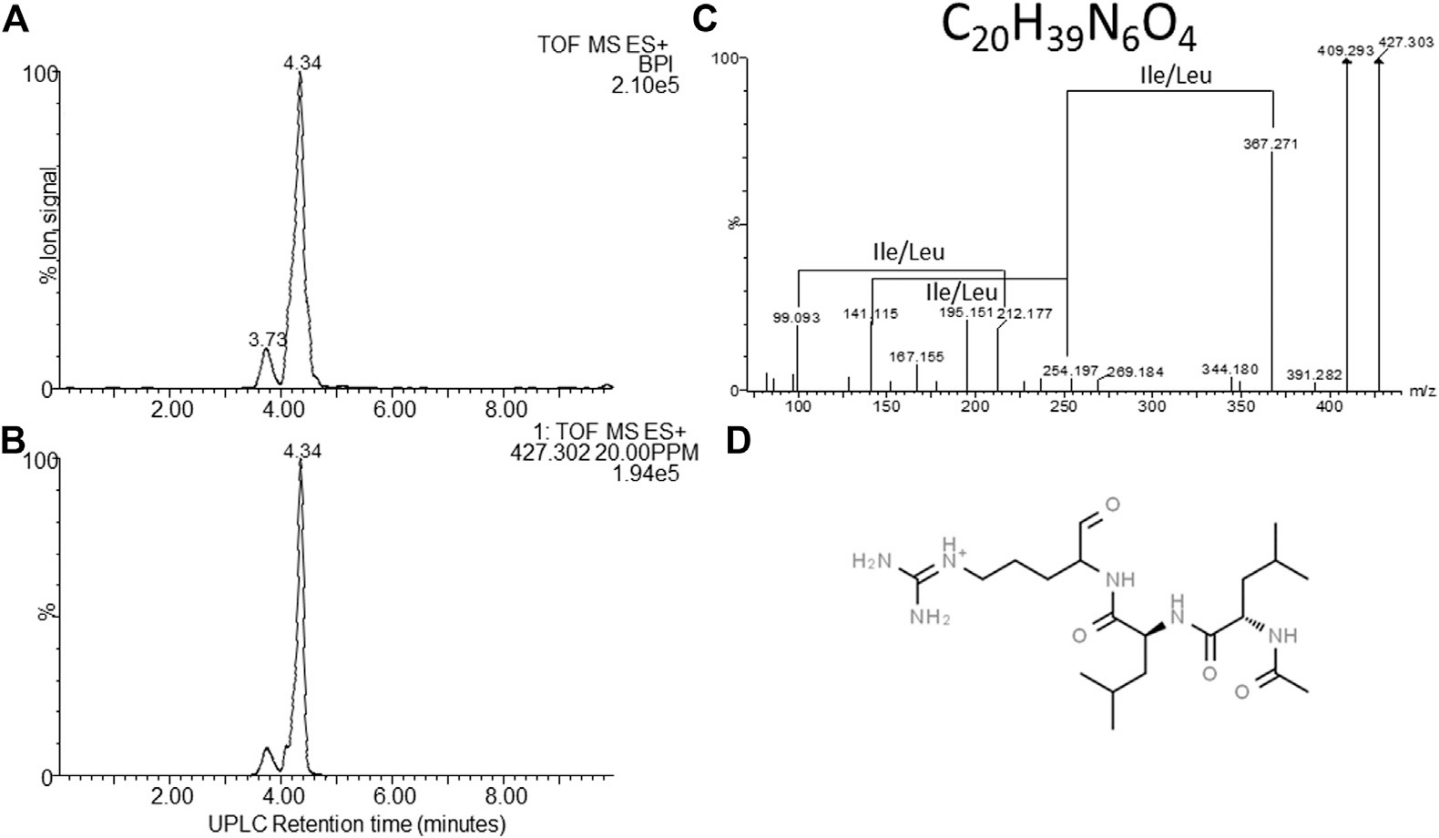
Structural analyses of compound **8** – khoicin. **(A)** UPLC-MS analysis of the purified compound and **(B)** the extracted *m/z* chromatogram of the khoicin ion with *m/z=* 427.302, **(C)** high resolution MS-MS spectrum of 427.302; **(D)** proposed working structure for khoicin.

**FIGURE 8:**
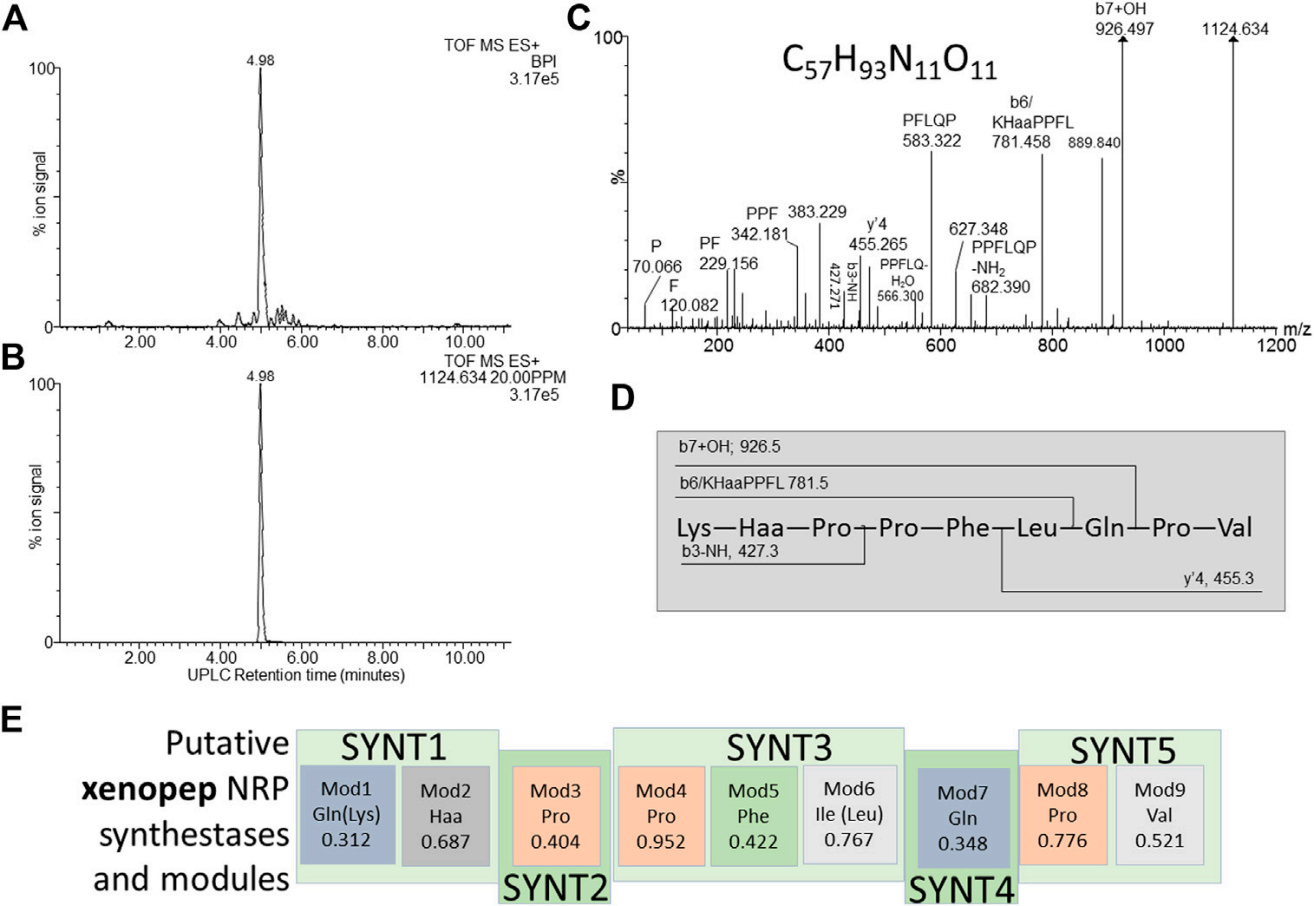
Structural analyses of compound **15** – xenopep. **(A)** UPLC-MS analysis of the purified compound and **(B)** the extracted *m/z* chromatogram of the xenopep ion with *m/z=* 1,124.364; **(C)** high resolution MS-MS spectrum of 1,124.364 annotated with putative ion identities; **(D)** proposed working structure for xenopep; **(E)** putative NRPS operon for xenopep with the predicted amino acids for each module (Mod) in the synthetase (SYNT) and probability score (the greater the number the lower the probability). The amino acids found with amino acid analysis and MS-MS are given in brackets.

The numbers within blocks indicate the relative signal in the ESMS detection of the different compounds (total sample signal was normalized and then the relative signal for each ion was calculated). The *m/z* values in bold indicated most abundant ions, those underlined were purified further for more detailed structural analyses (refer to Figures 5–8; Supplementary Figures S1–4) and known compounds and detected metabolites (refer to Figure 5; Table 3) are indicated with an * and #, respectively.

Over the first 6 min a diverse number of compounds were identified in the UPLC analysis of which 19 were selected for fingerprinting (Figure 3; Supplementary Figure S1 and Table 2). Similar polar compounds were found in NAO and SM extracts, while the profiles of NAA and AB correlated (Figure 3; Supplementary Figures S1, S2), with latter possibly due to the aqueous nature of the extract and culture media. The heatmap in Table 2 indicated the differences between the extracts. Notably the compounds observed with high ion intensity, 8 (*m/z* = 427.3), 16 (*m/z* = 889.5), 17 (*m/z* = 671.4), 18 (*m/z* = 407.2) were primarily found in the NAO and SM extracts. Only compounds 15 (*m/z* = 1124.6) is observed primarily in the more aqueous extracts, AB and NAA. We were able to purify compounds 8, 17, 15 and 18 for further study (refer to discussion below).

In the mid-gradient to late gradient (6–17 min) a plethora of small compounds are observed for the SM and less so for the NOA fraction (Figure 4; Supplementary Figures S3, S4 and Table 2). None of the compounds show a particularly high intensity in comparison with those eluting from 1–6 min of after 13 min.

The hydrophobic compounds (34–44) eluting after 13 min were all about 1.3 kDa with 14, 16 and 22 Da differences (Table 2), which indicated the possibly of a related peptide complex. We identified thee compounds, 39 (*m/z* = 1328.9) and 42 (*m/z* = 1314.8) with 44 (*m/z* = 1336.8) the Na-adduct of 42 for purification and further analysis. Refer to discussion below.

### Identification of Known Compounds

AntiSMASH identified 17 known biosynthetic gene clusters, of which eight were native to *Xenorhabdus* spp. (Figure 5). This presence of the xenocoumacin, xenoamicin and PAX peptide biosynthetic gene clusters led us to search for these compounds in the extracts.

Closer inspection of the compounds eluting from 1 to 6 min revealed compound 8 (*m/z* = 1,078.8) as PAX 7E in the NAA extract. We also found PAX1′, PAX3′ and PAX5 at lower levels in this extract **(** Figure 6B). The production of the PAX peptides in this *Xenorhabdus* strain was much lower than that found by Dreyer et al. (2019), and we were unable to purify any member of this family further. However, accurate mass analysis and the PAX biosynthetic gene cluster in this strain’s genome supported our identification (Table 3).

**TABLE 3:** Summary of previously identified secondary metabolites in the extracts of the three different *Xenorhabdus* cultures using high resolution UPLC-MS and MS analysis, as well as genetic analysis to confirm group identity. Refer to Figures 2–4; Table 2 for UPLC-MS and Supplementary Figures S1, S2 and S5 for high resolution MS analyses and structures.

Nr	Experimental *M*_r_		Proposed Identity	Theoretical *M*_r_		Mass error (ppm)	Comment/Reference
	Protonated	Sodiated		Protonated	Sodiated		
-	1050.7719	-	PAX5	1050.7766	-	4	Low concentration, Fuchs et al. (2011)
-	1052.7937	-	PAX1′	1052.7923	-	−1	Low concentration, Fuchs et al. (2011)
-	1066.8075	-	PAX3′	1066.8079	-	0.4	Low concentration, Fuchs et al. (2011)
8	1078.8058	-	PAX7E	1078.8079	-	2	Low concentration, Dreyer et al. (2019)
18	407.2171	-	Xenocoumacin II	407.2182	-	3	Reimer et al. (2011), Dreyer et al. (2019)
34	1286.8077	1308.7849	Xenoamicin D1	1286.8077	1308.7901	0, 4	Novel Xenoamicin? Zhou et al. (2013), this study
35, 36	1300.8235	1322.8052	Xenoamicin A1,2,3	1300.8238	1322.8058	0.2, 0.5	Novel Xenoamicins? Zhou et al. (2013), this study
37	1330.8326	-	Xenoamicin B2 +OH, x	1330.8344	-	1	Novel Xenoamicin, Zhou et al. (2013), this study
38, 39	1328.8552	1350.8311	Xenoamicin B1 (V→I/L)	1328.8552	1350.8371	0, 4	Novel Xenoamicin, Zhou et al. (2013), this study
42, 44	1314.8390	1336.8177	Xenoamicin B2 (I→V, I/L→V)	1314.8395	1336.8215	0.4, 3	Novel Xenoamicin, Zhou et al. (2013), this study

Compound 18 (*m/z* = 407.2) that eluted early at 3.71 min was observed in the NAO and SM fractions (Table 2). The compound displays a distinct yellow color and was purified and subsequently identified as xenocoumacin II (Figure 5; Supplementary Figure S6 and Table 3).

With the analysis of the late eluting compounds, we noted a compound range around 1.3 kDa that may be part of a peptide complex. As the xenoamicin biosynthetic gene cluster were found in this strain’s genome (Figure 5) this family was considered as possible candidate compounds. These peptide complexes were primarily identified in the SM fraction, although some of the members were also observed in the NAO fraction. We were able to co-purify compound 42 (*m/z* = 1314.8) and compound 38 (*m/z* = 1328.8) (Supplementary Figure S7; Table 2) and confirmed that both are members of the xenoamicin family (Figure 6; Supplementary Figure S7 and Table 3). However, the MS-MS structural analysis of compounds 38 and 42 showed a number of different fragments to that of xenoamicin G and B/C, respectively. Compound 38 shares the b1–b5 fragments with Xenoamicin B and X (Zhou et al., 2013) but differs by +14 Da in the observed y fragments. This indicated either a −CH_2_CH_3_ group in R_4_(Val→Ile) or iso/secondary butyl group in R_5_ (Val→Ile/Leu) (Figure 6; Supplementary Figure S7). Compound 38 was labeled as xenoamicin B2^v→I/L^. Compound 42 shares the b1–b4 fragments with Xenoamicin B and C (Zhou et al., 2013) but differs by −14 Da in b5 indicating a −CH_3_ group in R_3_(Ile→Val). Compound 42 also differed in the y’8 fragment by +14 Da. This indicated either a −CH_2_CH_3_ group in R_4_ (Val→Ile) or iso/secondary butyl group in R_5_ (Val→Ile/Leu) (Figure 6; Supplementary Figure S7). Compound 42 was labeled as xenoamicin B3^I→V, I/L→V^. Compound 37 (*m/z* = 1330.8), was also further characterized. This compound was labeled as a xenoamicin B1^+OH, x^ after its fragmentation pattern was compared with that of compound 42 (Supplementary Figure S8; Table 3). The MS-MS analysis indicated that the terminal group was hydroxylated while R_3_ group or residue was different from that of 42 and other reported xenoamicins. A number of other xenoamicins were also found in this peptide complex. Compound 34 with an identical Mr to xenoamicin D was denoted xenoamicin D1. Compound 35 and its sodium adduct (36) had three elution peaks (Table 2) indicating three different compounds with all three having an identical mass to xenoamicin A (Zhou et al., 2013) and was denoted xenoamicin A1,2,3 (Table 3). Three more compounds may be part of this complex, 40 (*m/z* = 1312.8), 41 (*m/z* = 1331.7) and 43 (*m/z* = 1352.8). This xenoamicin complex contains novel analogues that warrants future exploration.

### Novel Antimicrobial Compounds

The observed antibacterial activity of SM, NAA and NAO could be due to the PAX peptides and/or xenocoumacin II. However, other novel compounds could also contribute to this observed antibacterial activity. Furthermore, no known compounds with antibacterial activity were identified in the AB fraction, which could indicate that this fraction contains novel antimicrobial components. Twenty-one possible NRP synthetase operons were identified after utilizing the tblastn function of the NCBI website. This was further narrowed down to two putative operons by searching for the core motifs of the catalytic domains of the NRP synthetases. From this result we then predicted that at least two small non-ribosomally synthesized antimicrobial peptides or peptide analogues may be present in the extracts. Refer to Supplementary Figure S9 for the protein sequences of the two putative domains.

**FIGURE 9:**
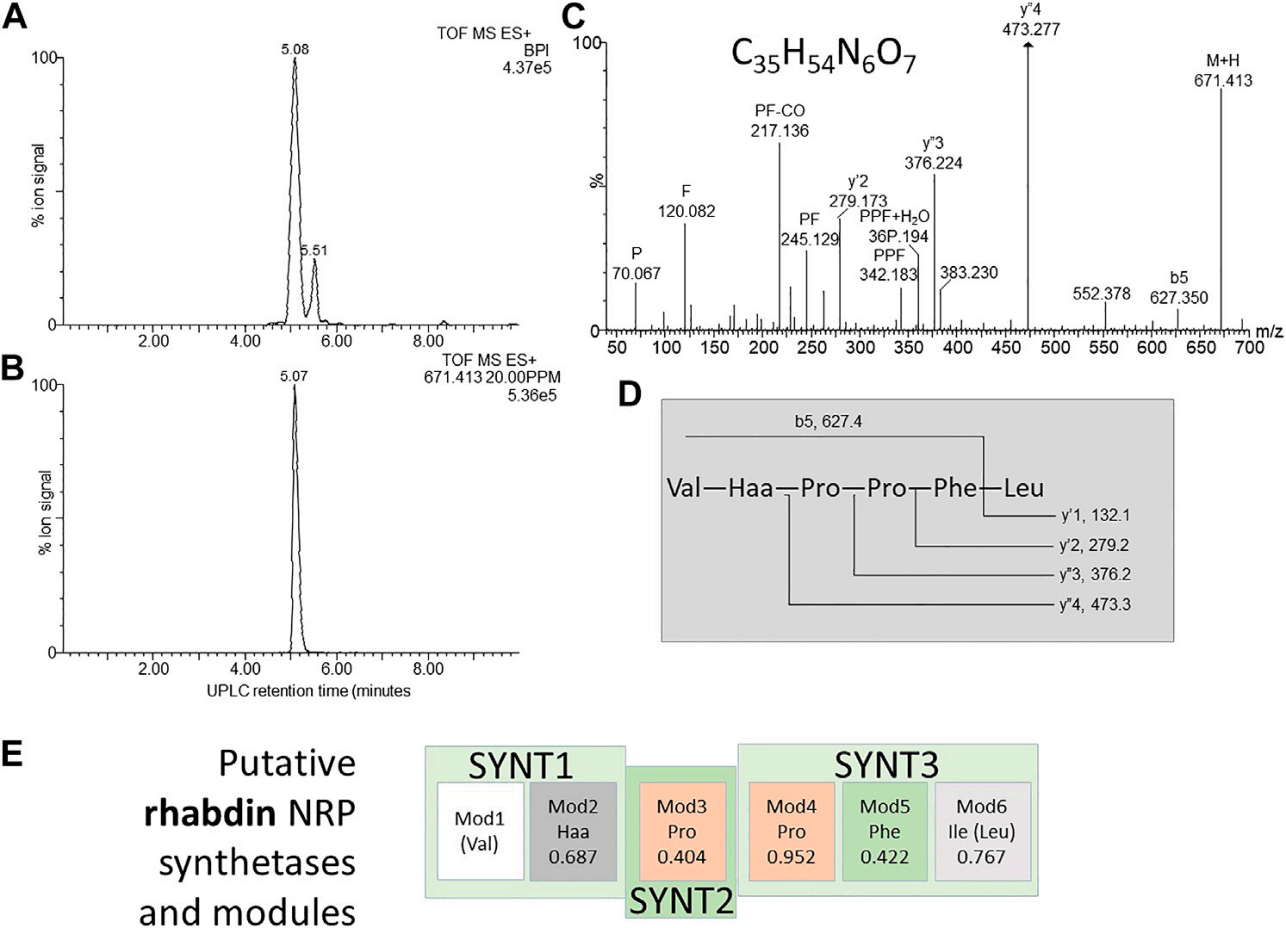
Structural analyses of compound **17** – rhabdin. **(A)** UPLC-MS analysis of the purified compound and **(B)** the extracted *m/z* chromatogram of the rhabdin ion with *m/z=* 671.413; **(C)** high resolution MS-MS spectrum of 671.413 annotated with putative ion identities; **(D)** proposed working structure for rhabdin; **(E)** putative NRPS operon for xenopep with the predicted amino acids for each module (Mod) in the synthetase (SYNT) and probability score (the greater the number the lower the probability). The amino acids found with amino acid analysis and MS-MS are given in brackets.

Three possible novel compounds and putative non-ribosomal antibacterial peptides namely compounds 8 (*m/z* = 427.3), 17 (*m/z* = 671.4) and 15 (*m/z* = 1124.6), were identified and purified from the various extracts (Figures 7–9). All three compounds were produced at appreciable amounts that assisted further purification to >75% purity (Figures 7–9). The antimicrobial activity of the three compounds were confirmed by screening the purified compounds against a library of bacterial targets (Table 4).

**TABLE 4:** Comparison of the antimicrobial activity spectrum of purified and partially purified novel compounds identified in the four *X. khoisanae* J194 extracts.

Compound number, proposed name	Activity against Gram-positive strains^a^		Activity against Gram-negative strains^a^	
8, Khoicin	*S. aureus* ATCC 25923 +++ *L. monocytogenes* +++ *S. epidermidis* +++ *B. subtilis* +	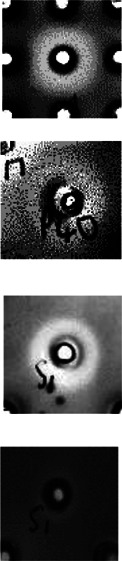	*E. coli* ++ *A. baumannii* +++ *K. pneumoniae* + *S. typhimurium* + *P. aeruginosa* ++	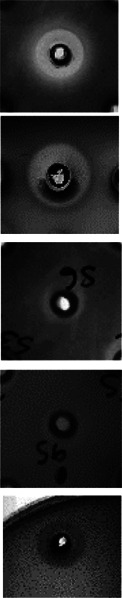
17, Rhabdin	*S. aureus* ATCC 25923 +++ *S. aureus* Xen 31 +++ *L. monocytogenes* +++	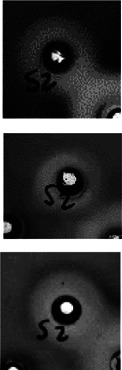	*K. pneumoniae* + *P. aeruginosa* ++ *E. coli* +	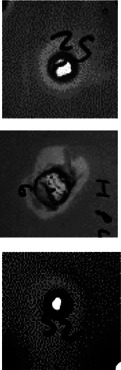
14, Xenopep	*S. aureus* ATCC 25923 + *L. monocytogenes* ++ *S. epidermidis* +++	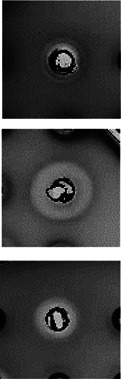	*Inactive*	NA




^a^ Activity +++ = very high, ++ = high, + = medium, if target cell of library tests (refer to methods) is not indicated, no activity was detected. The photos show the clear zones detected with the well diffusion assay with each of the respective target organisms.

### Compound 8–Khoicin

The first novel compound was named khoicin (8, *m/z* = 427.3) and exhibited a broad spectrum of potent Gram-positive and Gram-negative activity (Table 4). It is mainly produced in oxygen-limiting conditions and the highest amounts were extracted from a non-aerated broth culture (NAO) and a culture grown on solid media (SM). It was detected in the extracts in the following order: NAO>SM>AB>NAA (Figures 3B,D; Table 4). We determined the accurate mass of khoicin and derived its most probable elemental composition as C_20_H_39_N_6_O_4_. The MS-MS structural analysis of khoicin revealed that it fragmented into two major fragments, namely a dehydration product as *m/z* = 409.3 and a fragment ion at *m/z* = 367.3 which could be the result of the loss of an acetyl (CH_2_CO) group from the molecular ion (Figure 7). The rest of the fragment ions were at a much lower intensity. However, we could identify a fragment of 113.08 that indicated a Leu/Ile residue correlating with an immonium ion of Ile/Leu at *m/z* = 86.098. The fragments ions 254.197 and 227.101 could be mapped to an acetylated dipeptide containing Leu/Ile, respectively. One of the minor fragments at *m/z* = 156.113 mapped to Arg, which could explain the early elution of this compound. These results further indicate the peptide nature of khoicin and considering the elemental composition khoicin could consist of a formylated Arg linked to an acetylated Leu/Ile dipeptide, with Mr 426.2949, correlating with the experimental *M*_r_ of 426.2956 (−2 ppm mass error) (Figure 7).

### Compound 15–Xenopep

Compound 15, named, xenopep (*m/z* = 1124.6), was one of the early eluting peptide-like compounds primarily produced by aerated broth cultures (AB), suggesting that xenopep production could be oxygen dependent (Table 2). We were able to purify xenopep from AB cultures to >85% purity for further analysis (Figures 8A,B). Xenopep (16) is only active against Gram-positive bacteria (*S. aureus, S. epidermidis* and *L. monocytogenes*) and could thus be responsible for the inhibition of *S. epidermidis* and *L. monocytogenes* in AB and NAA extracts (Table 1 and Table 4). The purified peptide was subjected to amino acid analysis which revealed the amino acid composition to contain Val, Leu, Lys, Glu, Phe and three Pro residues.

We considered the two NRP synthetase operons to assist with the structure determination of xenopep, the first putative operon is located within node 7 and consist out of five genes encoding for the nine amino acids, with a predicted sequence of Gln-Haa-Pro-Pro-Ile-Gln-Pro-Val (Haa = hydrophobic amino acid) (Figure 8E). The sequence partially correlated with the determined amino acid composition. A conservative replacement of Ile with Leu improved this correlation, but one of the Gln residues must be replaced with Lys for a better correlation. The high-resolution MS-MS sequencing did not reveal a classical b- and y’’-ion pattern of a linear peptide acid but yielded a number of internal fragments (Figure 8C). Using the MS-MS analysis and predicted sequence from the xenopep putative operon, a working sequence was deduced as Lys-Haa-Pro-Pro-Leu-Gln-Pro-Val (Figure 8D), with Haa an undetermined hydrophobic residue.

### Compound 17–Rhabdin

Compound 17 (*m/z* = 671.4) was named rhabdin and its production was exclusive to the non-aerated broth (NAO) and SM (Table 2). This compound was purified to >80% (Figures 9A,B). Of the three early eluting peptide-like compounds, rhabdin is the second most active and inhibited the growth of *S. aureus, L. monocytogenes, K. pneumoniae, P. aeruginosa* and *E. coli* (Table 4).

Amino acid analysis conducted on rhabdin indicated the following amino acid composition: 2xPro, Val, Leu and Phe. his composition shares some similarities with xenopep. In the MS-MS structural analysis there was also significant correlation between the fragments observed for rhabdin and xenopep, with eight major fragments (*m/z* = 672.4, 383.2, 342.2, 245.1, 229.2, 217.1, 120.1, and 70.1) being identical (Figures 8C, 9C **)**. These fragments all map to the N-terminal part of xenopep. If the first three synthetases of the putative xenopep operon is used for rhabdin synthesis it would suggest the sequence as Val-Haa-Pro-Pro-Leu-Phe. We confirmed the sequence with MS-MS analysis, in which the fragmentation yielded classical y’’-ions suggesting a linear peptide acid (Figures 8C,D).

## Discussion

Bacteria from the genus *Xenorhabdus* are well known for their ability to produce a wide range of antimicrobial compounds, active against Gram-positive and -negative bacteria. Here we report on the activity of *X. khoisanae* J194 extracts against *P. aeruginosa, E. coli, S. aureus, S. epidermidis* and *L. monocytogenes* under different culture conditions. A previous study on *X. khoisanae* SB10 (Dreyer et al., 2019) also reported activity against *Bacillus subtilis* subsp. *subtilis* and *Candida albicans*. This suggests that some strains of *X. khoisanae* produce a range of antimicrobial compounds active against bacteria, yeast and possible also mycelial fungi.

The *in situ* analysis of the whole genome of *X. khoisanae* J194 confirmed the identification of xenocoumacin II, PAX peptides and xenoamicins. The analysis also gave insight into the sequence of two potential novel peptides, rhabdin and xenopep, identified in this study. The whole genome analysis gave additional support to the MS analysis that xenopep and rhabdin are novel but related peptides.

PAX 1′, 3′, 5 and 7E and xenocoumacin II were previously identified in cell-free extracts of *X. khoisanae* SB 10 (Dreyer et al., 2019). PAX 7E and PAX1′, 3′ and 5 are lipopeptides known for its activity against *Bacillus subtilis* BD170 and *E. coli* Xen 14 (Gualtieri et al., 2009; Dreyer et al., 2019) and could thus be present in fractions NAA and NAO. The second known compound, xenocoumacin II, is known for its antimicrobial and anti-ulcer activity. Xenocoumacin II is active against *S. aureus* and *E. coli,* as observed in studies on *X. nematophilia* (McInerney et al., 1991b) and *X. khoisanae* SB10 (Dreyer et al., 2019). It is thus safe to assume that xenocoumacin II, produced by *X. khoisanae* J194 and present in the NAA fraction, was below MIC levels required to kill *S. aureus* ATCC 25923. The presence of xenocoumacin II in the NAO and SM fractions could be responsible for the activity observed against *E. coli* Xen 14 and *S. aureus* ATCC 25923. The production of xenocoumacin in surface cultures and non-aerated broth indicated a role of oxygen in the control of its synthesis in this *X. khoisanae* strain and correlated with the results obtained by Dreyer et al. (2019). Due to its hydrophobic nature, the third family of compounds, xenoamicins, eluted much later (Zhou et al., 2013). We show that the xenoamicins in the produced complex exhibited some distinctly different fragments when compared to xenoamicin B/C and G described by Zhou et al. (2013), indicating that the xenoamicins produced by this strain might be novel. This is an important finding as xenoamicins are depsipeptides known for their anti-plasmodial activity (Zhou et al., 2013). However, they may not have contributed to the antimicrobial activity observed in NAO and SM fractions. Activity observed against *P. aeruginosa* ATCC 27853, *S. epidermidis* SE1 and *L. monocytogenes* EDGe is possibly due to novel compounds such as rhabdin and khoicin.

Khoicin and rhabdin are small early eluting compounds and provisionally characterized as a tri- and hexapeptide respectively, that are present in NAO and SM fractions. These could be responsible for inhibiting *P. aeruginosa* ATCC 27853, *S. epidermidis* SE1 and *L. monocytogenes* EDGe (Table 2 and Table 4). The size of both khoicin and rhabdin makes them ideal candidates for future antibiotics. The lack of known compounds identified in the AB fraction also indicated that *X. khoisanae* produces a plethora of possibly novel compounds, such as xenopep. Xenopep, a larger peptide also elutes early, but is primarily produced under aerated conditions, with a putative NRPS operon in node 7. The fact that a non-classical MS/MS fragmentation with internal fragments was observed could indicate that the peptide is modified and/or cyclic. Xenopep and rhabdin, share structural moieties, such as the sequence Pro-Pro-Leu-Phe. They may share an operon with differential expression with the synthetase 4 and 5, only expressed under anaerobic conditions to yield xenopep, while synthetases 1–3 are expressed under all conditions to yield rhabdin. The limited amounts of pure khoicin, rhabdin and xenopep precluded further structural analyses, which will be addressed in a future study.

Apart from the known compounds and the three novel compounds in this study an additional 28 compounds were detected with high ion signals. The bioactivity of these compounds is unknown and it is possible that strain J194 probably produce compounds with a wider bioactive profile than recorded in this study.

In an era where there is a dire need for new antibiotics, this study highlights the potential of discovering novel antibiotics from the rich complex of antimicrobials produced by *X. khoisanae* J194. The use of powerful techniques such as UPLC-MS and HPLC, combined with advanced microbiological techniques, proved to be a successful strategy. Further research is required to determine the full antimicrobial potential of rhabdin and khoicin. This study also showed the immense influence aeration have on the secondary metabolism of *X. khoisanae* J194. Indicating that researchers should investigate a more diverse range of conditions when mining for novel antibiotics from microorganisms. The variation in antimicrobial compounds produced by *X. khoisanae* J194 may lead to the discovery of novel antibiotics.

## Materials and Methods

### Isolation, Stock and Growth Conditions of Strains

*Xenorhabdus khoisanae* J194 was isolated from the nematode *Steinernema jeffreyense* J194 (Dreyer et al., 2017), collected from soil in the Eastern Cape, South Africa (Malan et al., 2016)*.* Cells of *X. khoisanae* J194 were plated onto nutrient agar, supplemented with 0.025% (w/v) bromothymol blue and 0.04% (*w/v*) TTC (NTBA), and incubated at 30°C for a minimum of 48 h. Blue colonies, representative of cells in phase I (Dreyer et al., 2017), were selected and cultured in Tryptone Soy Broth (TSB) at 37°C for 24 h on an orbital shaker. Pure colonies were stored at −80°C in the presence of 40% (*v/v*) sterile glycerol. *Staphylococcus aureus* ATCC 25923, *S. aureus* Xen 31, *S. epidermidis* SE1, *L. monocytogenes* EDGe, *E. coli* Xen 14 and *P. aeruginosa* ATCC 27853 were from the culture collection of the Department of Microbiology, Stellenbosch University, and were grown in Brain Heart Infusion (BHI). These strains were streaked to purity on BHI Agar. Colonies were resuspended into sterile phosphate buffer (PBS), transferred to 80% (*v/v*) sterile glycerol (final 40%, *v/v*) and stored at −80°C.

### Isolation of Antimicrobial Compounds

*Xenorhabdus khoisanae* J194 was cultured in TSB for 24 h at 30°C and then streaked onto Tryptone Soy Agar (TSA). Colonies were suspended in 5 mL TSB and used as inoculum in each of the three following culturing methods.

In method A, 10 mL of TSB in a 100 mL Erlenmeyer flask was inoculated with 100 μL cell suspension and incubated at 26°C for 24, 48, 72 and 96 h, respectively. At each of these time points, 1.0 ml was sampled to record the pH and optical density (OD_595_). The pH was of each sample was adjusted to 4.0 with 10.2 M HCl and kept at 4°C for 3 h. Cells were harvested (5,000 × *g*, 20 min, 4°C) and the cell-free supernatants (CFSs) concentrated by freeze-drying. Freeze-dried samples were re-suspended, separately, in 10 mL 75% (*v/v*) acetonitrile. The acetonitrile upper fraction and oily bottom fraction were separated by carefully removing the top layer with a pipette and freeze-dried. Harvested cells were resuspended in 10 mL 90% (*v/v*) acetonitrile to extract intracellular antimicrobial compounds, vortexed and centrifuged (5,000 × *g*, for 20 min, 4°C) to remove cell debris. Cell-free supernatants were concentrated by freeze-drying.

In method B, 250 mL TSB, in a 250 mL Erlenmeyer flask was inoculated with 2.5 mL active growing cell suspension and incubated at 26°C for 24, 48, 72 and 96 h, respectively, on an orbital shaker (220 rpm). Samples (1.0 mL) were collected after 24, 48, 72 and 96 h, and the pH and OD readings determined as described in method A. At each time point, cells were harvested, as described above, 10 g Amberlite XAD-16 resin (Sigma-Aldrich, Missouri, United States) were added to 250 mL cell-free supernatant (CFS) and left on an orbital shaker (180 rpm) at 4°C for 3 h. The beads were removed by filtration, washed with 1 L of double distilled water and incubated with 30% (*v/v*) ethanol (25 mL/5 g) for 30 min to remove weakly bound polar compounds. After 30 min the beads were collected by filtration and ethanol was washed of with 1 L of double distilled water. The more hydrophobic and amphipathic compounds were released from the beads by incubating the beads with 80% (*v/v*) isopropanol containing 0.1% (*v/v*) TFA (trifluoroacetic acid) (ISO-TFA; 40 mL per 5.0 g) for 1 h. The 80% isopropanol and beads were separated by filtration and the isopropanol was removed under vacuum, using a Rotavapor R-114 (Büchi) connected to a water bath (B-480, Büchi). Further concentration was accomplished by freeze-drying.

In method C, 5.0 mL cell suspension was mixed with 5.0 g sterile Amberlite XAD-16 resin and then plated onto TSA (TSB with 2.0%, *w/v*, agar) in four sets of 150 mm diameter plates. TSB was pre-treated with Amberlite XAD-16 resin to remove most of the hydrophobic compounds before agar was added. Plates were incubated at 26°C for 24, 48, 72 and 96 h, respectively. At each of these time points, the Amberlite XAD-16 resin were removed from the surface of the agar with a sterile metal scraper, added to 200 mL analytical quality water and agitated on an orbital shaker (180 rpm) at 4°C for 1 h. The beads were then collected and incubated with 30% (*v/v*) ethanol (25 mL per 5.0 g) as described above to remove polar compounds. After 30 min the hydrophobic and amphipathic compounds were released from the Amberlite XAD-16 resin by incubating the resin in 80% (*v/v*) isopropanol, containing 0.1% (*v/v*) TFA (ISO-TFA; 40 mL per 5.0 g) for 1 h. The isopropanol and resin were separated as described above and the isopropanol was removed by evaporation and the hydrophobic and amphipathic fraction concentrated by freeze-drying, as described elsewhere.

### Antimicrobial Activity Tests

Overnight-grown cultures of *S. aureus* ATCC 25923, *L. monocytogenes* EDGe, *E. coli* Xen 14 and *P. aeruginosa* ATCC 27853 were inoculated (1.0%, *v/v*) into separate volumes of sterile molten TSA (approximately 45°C), carefully swirled, immediately transferred to wells (100 µL per well) in a 96-well microtiter plate (Greiner Bio-One, Austria) and left to solidify. Refer to Du Toit and Rautenbach (2000) for a more detailed description. Freeze-dried fractions were each suspended in 50% (*v/v*) acetonitrile in water to yield a final volume of 1.0 mL. Twenty microliters of each suspension of the freeze-dried crude extracts (24, 48, 72, and 96 h) were added onto the surface of TSA-imbedded cells in the 96-well plate and the plate was incubated for 24 h at 37°C. Optical density readings (595 nm) were recorded for each well before and after 24 h of incubation at 37°C, using an iMark Microplate reader (Biorad, California, United States).

Activity assays of the 96 h crude extracts were repeated to determine specific activity. Freeze-dried CFS collected from 96 h-old cultures were each re-suspended in sterile distilled water (4.0 mg/mL) and antimicrobial activity tested against *P. aeruginosa* ATCC 27853, *E. coli* Xen 14, *S. aureus* ATCC 25923, *S. aureus* Xen 31, *S. epidermidis* SE1 and *L. monocytogenes* EDGe. The strains were inoculated (1.0%, *v/v*) into separate volumes of sterile molten TSA (approximately 45°C), carefully mixed and immediately transferred to round petri dishes of 145 mm in diameter in the first step of the well diffusion agar assay. A 96-well PCR plate was sterilized under UV, placed into the inoculated molten agar and the agar was allowed to set before the PCR plate were removed. Once the agar was set, 20.0 µL crude extract was placed into each well and incubated for 24 h at 37°C. The same method was used to determine the antimicrobial activity of the partially purified compounds, except all the fractions collected were dissolved in 100 µL of 50% acetonitrile. Specific activity was determined using the following equation:

$$Specific\;activity=\frac{area/mL}{4.00\;\;mg/mL}$$

### *In Situ* Identification of Putative Operons

The whole genome of *X. khoisanae* J194 was subjected to AntiSMASH (Antismash.secondarymetabolites.org) to identify secondary metabolites biosynthesis gene clusters (Blin et al., 2019). The whole genome was also analyzed for putative operons relating to rhabdin and xenopep. Operons were identified by aligning the protein sequence of gramicidin S and tyrocidine synthetases to the whole genome sequence of *X. khoisanae* J194 using the tblastn function of the NCBI website (blast.ncbi.nlm.nih.gov/Blast.cgi). The open reading frames of the possible operons were translated to protein sequence using the CLC Main Workbench 7.6.1 software. Thereafter the various domains present in non-ribosomal peptide (NRP) synthetases (condensation, adenylation, epimerization, thiolation and thioesterase) were identified by searching for the highly conserved core motifs of the catalytic NRP domains. The synthetases identified were subjected to the LSI based A-domain function predictor (http://bioserv7.bioinfo.pbf.hr/LSIpredictor/AdomainPrediction.jsp), the NRPSsp adenylation domain predictor (http://www.nrpssp.com) and the PKS/NRPS Web Server/Predictive Blast Server (http://sourceforge.net/projects/secmetdb/). Validity of the prediction was based on the prediction score given by the software.

### Partial Purification of Antimicrobial Compounds

Compounds produced by *X. khoisanae* J194 cultured on solid media were extracted and partially purified by means of high-performance liquid chromatography (HPLC; Agilent, California, United States). The freeze-dried extract was dissolved in 50% (*v/v*) acetonitrile and loaded onto a Poroshell 120 EC-C_18_ HPLC column (120 Å, 4 μm, 4.6 mm × 150 mm, Agilent) and eluted with a linear gradient created with 0.1% (*v/v*) trifluoracetic acid (TFA) in analytically pure water (eluent A) and 0.1% (*v/v*) TFA in acetonitrile (eluent B). The flow rate was set at 1.5 mL/min and the elution program utilized was as follows: 20% eluent A from 0 to 0.5 min (initial conditions), 0.5–12.5 min linear gradient from 20 to 90% eluent B, 12.5–13 min linear gradient from 90 to 100% eluent B, 13–14 min at 100% eluent B, followed by column equilibration (14–20 min linear gradient from 100 to 20% eluent B and 20–25 min at initial conditions). Separation was performed on an Agilent 1260 Infinity II LC system. Data were recorded at 230 and 245 nm. Fractions of the peaks were collected, freeze-dried and tested for antimicrobial activity as described above.

### Identification of Antimicrobial Compounds

Samples that were active against one or more of the target strains were analyzed using a Waters Acquity UPLC™ (ultraperformance liquid chromatography), linked to a Waters Synapt G2 quadrupole time-of-flight mass spectrometer (QTOF; Waters Corporation, Milford, United States) housed at the Central Analytical Facility at Stellenbosch University. Sample volumes of 1.0–5.0 µL were analyzed on a HSS T3 column (1.8 µm, 2.1 mm × 100 mm) developed with an elution program created with 0.1% (*v/v*) formic acid in analytically pure water (eluent A) and 0.1% (*v/v*) formic acid in acetonitrile (eluent B). The flow rate was set at 300 µL/min and the elution program utilized was as follows: 100% eluent A from 0 to 0.5 min (initial conditions), 0.5–1.0 min linear gradient from 0 to 30.0% eluent B, 1.0–10.0 min linear gradient from 30.0 to 60.0% eluent B, 10–15 min linear gradient from 60.0 to 80.0% eluent B, followed by column equilibration (15.0–15.1 min linear gradient from 80.0 B to 100% A and 15.1–20.0 min at initial conditions). The rest of the instrument settings for mass spectrometric analysis were as follows: cone voltage 15 V, capillary voltage 2.5 kV, extraction cone voltage 4 V, source temperature 120°C, desolvation gas (N_2_) of 650 L/h and temperature 275°C. Spectral data were collected in positive mode by scanning through *m/z* = 100 to 2000 in continuum mode. Mass-spectrometry data of the detected ion peaks in chromatography of the different samples were processed with the MaxEnt 3 function of MassLynx 4.1. For comparison of samples, signals were normalized to the highest signal over a specific elution range (0–6 min and 6–17 min).

Preliminary structure analysis was done by high resolution collision induced dissociation (CID) analyses in the MS^E^ mode (tandem MS or MS/MS) during UHPLC-MS and monitored on a second MS channel, as described by Dreyer et al. (2019). A collision energy gradient of 20–60 eV at 1 s MS/MS scan time were used for CID and data was collected in the second mass analyser (MS2) through *m/z* = 40 to 2000 in continuum mode. Reliable high-resolution MS data was ensured by calibrating the MS instrument with sodium formate. A single point lock spray (leucine encephalin, *m/z* = 556.2771) was used as a calibrant during analysis to compensate for any *m/z* drift. Elemental composition was fitted using Masslynx software and formulas were considered that has a mass accuracy of 5 ppm or better. The elution time, number of hetero atoms, isotope patterns and double bond equivalence were taken into consideration when probable formulas (elementary composition) were selected. Candidate formulas were not selected for larger molecules, due to high number of possibilities, and therefore a high uncertainty, unless they were previously identified compounds.

## Data Availability

The raw data supporting the conclusions of this article are available in Supplementary Material. Any other data will be made available by the authors, without undue reservation.

## References

[B1] Arbeit R. Maki D. Tally F. O. Campanaro E. Eisenstein B. I. (2004). The safety and efficacy of daptomycin for the treatment of complicated skin and skin-structure infections. Clin Infect Dis. 38, 1673–1681. 10.1086/420818 15227611

[B2] Blin K. Shaw S. Steinke K. Villebro R. Ziemert N. Lee S. Y. (2019). antiSMASH 5.0: updates to the secondary metabolite genome mining pipeline. Nucleic Acids Res. 47, W81–W87. 10.1093/nar/gkz310 31032519 PMC6602434

[B3] Booysen E. Dicks L. M. T. (2020). Does the future of antibiotics lie in secondary metabolites produced by *Xenorhabdus* spp.? *A Review* . Probiotics Antimicrob. Proteins 12, 1310–1320. 10.1007/s12602-020-09688-x 32844362

[B4] Böszörményi E. Érsek T. Fodor A. M. Fodor A. M. Földes L. S. Hevesi M. (2009). Isolation and activity of *Xenorhabdus* antimicrobial compounds against the plant pathogens *Erwinia amylovora* and *Phytophthora nicotianae* . J. Appl. Microbiol. 107, 746–759. 10.1111/j.1365-2672.2009.04249.x 19320949

[B5] Cai X. Challinor V. L. Zhao L. Reimer D. Adihou H. Grün P. (2017). Biosynthesis of the antibiotic nematophin and its elongated derivatives in entomopathogenic bacteria. Org. Lett. 19, 806–809. 10.1021/acs.orglett.6b03796 28134534

[B6] Chen G. Maxwell P. Dunphy G. B. Webster J. M. (1996). Culture conditions for *Xenorhabdus* and *Photorhabdus* symbionts of entomopathogenic nematodes. Nematologica 42, 127–130. 10.1163/187529296x00139

[B7] Crawford J. M. Portmann C. Kontnik R. Walsh C. T. Clardy J. (2011). NRPS substrate promiscuity diversifies the xenematides. Org. Lett. 13, 5144–5147. 10.1021/ol2020237 21888371 PMC3184645

[B8] Crawford J. M. Portmann C. Zhang X. Roeffaers M. B. J. Clardy J. (2012). Small molecule perimeter defense in entomopathogenic bacteria. Proc. Natl. Acad. Sci. U. S. A. 109, 10821–10826. 10.1073/pnas.1201160109 22711807 PMC3390839

[B9] Crawford J. M. Kontnik R. Clardy J. (2010). Regulating alternative lifestyles in entomopathogenic bacteria. Curr. Biol. 20, 69–74. 10.1016/j.cub.2009.10.059 20022247 PMC2821981

[B10] D’Costa V. M. King C. E. Kalan L. Morar M. Sung W. W. L. Schwarz C. (2011). Antibiotic resistance is ancient. Nature 477, 457–461. 10.1038/nature10388 21881561

[B11] Dreyer J. Malan A. P. Dicks L. M. T. (2018). Bacteria of the genus X*enorhabdus*, a novel source of bioactive compounds. Front. Microbiol. 9, 1–14. 10.3389/fmicb.2018.03177 30619229 PMC6305712

[B12] Dreyer J. Malan A. P. Dicks L. M. T. (2017). Three novel *Xenorhabdus* – *Steinernema* associations and evidence of strains of *X. khoisanae* switching between different clades. Curr. Microbiol. 74, 938–942. 10.1007/s00284-017-1266-2 28526895

[B13] Dreyer J. Rautenbach M. Booysen E. Van Staden A. D. Deane S. M. Dicks L. M. T. (2019). *Xenorhabdus khoisanae* SB10 produces Lys-rich PAX lipopeptides and a Xenocoumacin in its antimicrobial complex. BMC Microbiol. 19, 1–11. 10.1186/s12866-019-1503-x 31195965 PMC6567599

[B14] Du Toit E. A. Rautenbach M. (2000). A sensitive standardised micro-gel well diffusion assay for the determination of antimicrobial activity. J. Microbiol. Methods 42, 159–165. 10.1016/S0167-7012(00)00184-6 11018272

[B15] Dunphy G. B. Webster J. M. (1991). Antihemocytic surface components of *Xenorhabdus nematophilus* var*. dutki* and their modification by serum nonimmune larvae of *Galleria mellonella* . J. Invertebr. Pathol. 58, 40–51. 10.1016/0022-2011(91)90160-r

[B16] Fair R. J. Tor Y. (2014). Antibiotics and bacterial resistance in the 21st century. Perspect. Medicin. Chem. 6, 25–64. 10.4137/PMC.S14459 25232278 PMC4159373

[B17] Ferreira T. van Reenen C. A. Endo A. Spröer C. Malan A. P. Dicks L. M. T. (2013). Description of *Xenorhabdus khoisanae* sp. nov., the symbiont of the entomopathogenic nematode *Steinernema khoisanae* . Int. J. Syst. Evol. Microbiol. 63, 3220–3224. 10.1099/ijs.0.049049-0 23456807

[B18] Fuchs S. W. Grundmann F. Kurz M. Kaiser M. Bode H. B. (2014). Fabclavines: bioactive peptide – polyketide-polyamino hybrids from *Xenorhabdus* . Chembiochem. 15, 512–516. 10.1002/cbic.201300802 24532262

[B19] Fuchs S. W. Proschak A. Jaskolla T. W. Karas M. Bode H. B. (2011). Structure elucidation and biosynthesis of lysine-rich cyclic peptides *in Xenorhabdus namtophila* . Org. Biomol. Chem. 9, 3130–3132. 10.1039/c1ob05097d 21423922

[B20] Grundmann F. Kaiser M. Kurz M. Schiell M. Batzer A. Bode H. B. (2013). Structure determination of the bioactive depsipeptide xenobactin from *Xenorhabdus* sp. PB30.3. RSC Adv. 3, 22072–22077. 10.1039/c3ra44721a

[B21] Gualtieri M. Aumelas A. Thaler J.-O. (2009). Identification of a new antimicrobial lysine-rich cyclolipopeptide family from *Xenorhabdus nematophila* . *J. Antibiot*. (Tokyo). 62, 295–302. 10.1038/ja.2009.31 19373275

[B22] Guo S. Zhang S. Fang X. Liu Q. Gao J. Bilal M. (2017). Regulation of antimicrobial activity and xenocoumacins biosynthesis by pH in *Xenorhabdus nematophila* . Microb. Cell Fact. 16, 1–14. 10.1186/s12934-017-0813-7 29141647 PMC5688692

[B23] Hacker C. Cai X. Kegler C. Zhao L. Weickhmann A. K. Wurm J. P. (2018). Structure-based redesign of docking domain interactions modulates the product spectrum of a rhabdopeptide-synthesizing NRPS. Nat. Commun. 9, 1–11. 10.1038/s41467-018-06712-1 30341296 PMC6195595

[B24] Ji D. Yi Y. Kang G. H. Choi Y. H. Kim P. Baek N. I. (2004). Identification of an antibacterial compound, benzylideneacetone, from *Xenorhabdus nematophila* against major plant-pathogenic bacteria. FEMS Microbiol. Lett. 239, 241–248. 10.1016/j.femsle.2004.08.041 15476972

[B25] Kaufman G. (2011). Antibiotics: mode of action and mechanisms of resistance. Nurs. Stand. 24, 49–55. 10.7748/ns.25.42.49.s52 21826872

[B26] Kronenwerth M. Bozhüyük K. A. J. Kahnt A. S. Steinhilber D. Gaudriault S. Kaiser M. (2014). Characterisation of taxlllaids A-G; natural products *from Xenorhabdus indica* . Chem. - A Eur. J. 20, 17478–17487. 10.1002/chem.201403979 25351611

[B27] Lee Ventola C. (2015). The antibiotic resistance crisis: Part 2: Management strategies and new agents. Pharm. Ther. 40, 344–352. 10.7416/ai.2019.2253 PMC4422635 25987823

[B28] Li B. Wever W. J. Walsh C. T. Bowers A. A. (2014). Dithiolopyrrolones: biosynthesis, synthesis and activity of a unique class of disulfide containing antibiotics. Nat. Prod. Rep. 31, 905–923. 10.1039/c3np70106a 24835149 PMC4132845

[B29] Malan A. P. Knoetze R. Tiedt L. R. (2016). *Steinernema jeffreyense* n. Sp. (Rhabditida: Steinernematidae), a new entomopathogenic nematode from South Africa. J. Helminthol. 90, 262–278. 10.1017/S0022149X15000097 25758326

[B31] McInerney B. V. Gregson R. P. Lacey M. J. Akhurst R. J. Lyons G. R. Rhodes S. H. (1991a). Biologically active metabolites from *Xenorhabdus* spp., Part 1. Dithiolopyrrolone derivatives with antibiotic activity. J. Nat. Prod. 54, 774–784. 10.1021/np50075a005 1955880

[B32] McInerney B. V. Taylor W. C. Lacey M. J. Akhurst R. J. Gregson R. P. (1991b). Biologically active metabolites from *Xenorhabdus* spp., Part 2. Benzopyran-1-one Derivatives with Gastroprotective activity. J. Nat. Prod. 54, 785–795. 10.1021/np50075a006 1955881

[B33] Park D. Ciezki K. van der Hoeven R. Singh S. Reimer D. Bode H. B. (2009). Genetic analysis of xenocoumacin antibiotic production in the mutualistic bacterium *Xenorhabdus nematophila* . Mol. Microbiol. 73, 938–949. 10.1111/j.1365-2958.2009.06817.x 19682255

[B34] Proschak A. Zhou Q. Schöner T. Thanwisai A. Kresovic D. Dowling A. (2014). Biosynthesis of the insecticidal xenocyloins in *Xenorhabdus bovienii* . Chembiochem 15, 369–372. 10.1002/cbic.201300694 24488732

[B35] Qin Z. Huang S. Yu Y. Deng H. (2013). Dithiolopyrrolone natural products: isolation, synthesis and biosynthesis. Mar. Drugs 11, 3970–3997. 10.3390/md11103970 24141227 PMC3826145

[B36] Reimer D. Nollmann F. I. Schultz K. Kaiser M. Bode H. B. (2014). Xenortide biosynthesis by entomopathogenic *Xenorhabdus nematophila* . J. Nat. Prod. 77, 1976–1980. 10.1021/np500390b 25080196

[B37] Reimer D. Pos K. M. Thines M. Grün P. Bode H. B. (2011). A natural prodrug activation mechanism in nonribosomal peptide synthesis. Nat. Chem. Biol. 7, 888–890. 10.1038/nchembio.688 21926994

[B38] Reimer D. Luxenberger E. Brachmann A. O. Bode H. B. (2009). A new type of pyrrolidine biosynthesis is involved in the late steps of xenocoumacin production in *Xenorhabdus nematophila* . Chembiochem 10, 1997–2001. 10.1002/cbic.200900187 19598185

[B39] Singh J. Banerjee N. (2008). Transcriptional analysis and functional characterization of a gene pair encoding iron-regulated xenocin and immunity proteins of *Xenorhabdus nematophila* . J. Bacteriol. 190, 3877–3885. 10.1128/JB.00209-08 18375563 PMC2395030

[B40] Spellberg B. Guidos R. Gilbert D. Bradley J. Boucher H. W. Scheld M. W. (2008). The epidemic of antibiotic-resistant infections: a call to action for the medical community from the infectious diseases society of America. Clin. Infect. Dis. 46, 155–164. 10.1086/524891 18171244

[B41] Thaler J. O. Baghdiguian S. Boemare N. (1995). Purification and characterization of xenorhabdicin, a phage tail-like bacteriocin, from the lysogenic strain F1 of *Xenorhabdus nematophilus* . Appl. Environ. Microbiol. 61, 2049–2052. 10.1128/AEM.61.5.2049-2052.1995 7646048 PMC167475

[B42] Ventola C. L. (2015). Antibiotic Resistance Crisis: part 1: causes and threats. Pharm. Ther. 40, 277–283. 10.24911/ijmdc.51-1549060699 PMC4378521 25859123

[B43] Wang Y. H. Feng J. T. Zhang Q. Zhang X. (2008a). Optimization of fermentation condition for antibiotic production by *Xenorhabdus nematophila* with response surface methodology. J. Appl. Microbiol. 104, 735–744. 10.1111/j.1365-2672.2007.03599.x 17953686

[B44] Wang Y. H. Li Y. P. Zhang Q. Zhang X. (2008b). Enhanced antibiotic activity of *Xenorhabdus nematophila* by medium optimization. Bioresour. Technol. 99, 1708–1715. 10.1016/j.biortech.2007.03.053 17531470

[B45] Wang Y. Fang X. An F. Wang G. Zhang X. (2011a). Improvement of antibiotic activity of *Xenorhabdus bovienii* by medium optimization using response surface methodology. Microb. Cell Fact. 10, 98. 10.1186/1475-2859-10-98 22082189 PMC3227641

[B46] Wang Y. Fang X. Cheng Y. Zhang X. (2011b). Manipulation of pH shift to enhance the growth and antibiotic activity of *Xenorhabdus nematophila* . J. Biomed. Biotechnol. 2011, 672369. 10.1155/2011/672369 21660139 PMC3110314

[B47] Webster J. M. Li J. Chen G. (1996). Xenorxides with antibacterial and antimycotic properties. WO1996032396A1. 1–12.

[B48] Yang J. Zeng H.-M. Lin H.-F. Yang X.-F. Liu Z. Guo L.-H. (2012). An insecticidal protein from *Xenorhabdus budapestensis* that results in prophenoloxidase activation in the wax moth, *Galleria mellonella* . J. Invertebr. Pathol. 110, 60–67. 10.1016/j.jip.2012.02.006 22387345

[B49] Zavascki A. P. Goldani L. Z. Li J. Nation R. L. (2007). Polymyxin B for the treatment of multidrug-resistant pathogens: a critical review. J. Antimicrob. Chemother. 60, 1206–1215. 10.1093/jac/dkm357 17878146

[B50] Zhou Q. Grundmann F. Kaiser M. Schiell M. Gaudriault S. Batzer A. (2013). Structure and biosynthesis of xenoamicins from entomopathogenic *Xenorhabdus* . Chem. Eur. J. 19, 16772–16779. 10.1002/chem.201302481 24203528

[B51] Zhou L. Kaiser M. Bode H. (2018). Rhabdopeptide/xenortide-like peptides from *Xenorhabdus innexi* with terminal amines showing potent antiprotozoal activity. Organic Lett. 20, 5116–5120. 10.1021/acs.orglett.8b01975 30095261

