# Supplementary material for: Profiling the Production of Antimicrobial Secondary Metabolites by Xenorhabdus khoisanae J194 Under Different Culturing Conditions
Source: Front Chem. 2021 Mar 30;9:626653. doi: 10.3389/fchem.2021.626653 (PMC8042232; doi:10.3389/fchem.2021.626653)
Supplement: Supplementary file 1 [file datasheet1.docx]

**SUPPLEMENTARY DATA**

**Profiling the production of antimicrobial secondary metabolites by *Xenorhabdus khoisanae* J194 under different culturing conditions**

*Elzaan Booysen^1^, Marina Rautenbach^2^*, Marietjie A Stander^3,4^, Leon MT Dicks^1^**

^1^Department of Microbiology, ^2^Department of Biochemistry, ^3^LCMS Central Analytical Facility, Stellenbosch University, Stellenbosch, South Africa; *correspondence must be directed to Marina Rautenbach - [mra@sun.ac.za](mailto:mra@sun.ac.za); Leon Dicks - [LMTD@sun.ac.za](mailto:LMTD@sun.ac.za)

**UPLC-MS/UV fingerprinting of *Xenorhabdus khoisanae* J194 culture extracts**

Figs. S1 and S2 depict the UPLC profiles of four culture extracts of aqueous fraction of non-aerated broth culture (NAA), fraction of non-aerated broth culture, (NAO), extract from aerated broth culture and (AB) and extract from culture grown on surface of solid medium( SM). over the first 6 minute. of chromatography. The linked mass spectra and UV spectra of the selected UPLC peaks is also supplied. The ions detected first 6 minutes represent the most polar and overt amphipathic compounds in the extract.

Figs. S3 and S4 depict the UPLC profiles of four culture extracts of NAO and SM over the mid gradient of 6-13 minutes of chromatography. The linked mass spectra and UV spectra of the selected UPLC peaks is also supplied. The ions detected from 6-13 minutes represent the amphipathic compounds in the extract.

Figs. S5 depicts the UPLC profiles of four culture extracts NAA, NAO, AB and SM over the late gradient of 13-17 minutes of chromatography. The linked mass spectra of the selected UPLC peaks are also supplied. These compounds did not show any appreciable UV spectrum apart from absorption above 240 nm (results not shown). The ions detected from 13-17 minutes represent the more hydrophobic compounds in the extract.

Compounds ion that was selected are indicated in bold, while those indicated with * represent non-covalent dimers of the main ion (+1) that were detected.

Figs. S6 depicts the structural analyses of compound **18** – xenocoumacin II. UPLC-MS analysis of the purified compound and the extracted *m/z* chromatogram of the xenocoumacin II ion with *m/z=* 407.210 and the high-resolution MS-MS spectrum of 407.210 can be seen in this figure, as well as the Uv spectrum of the xenocoumacin II.

Figs. S7 depicts the structural analyses of compounds **38** and **42** – xenoamicins. UPLC-MS-MS analysis showing the m/z extracted chromatogram of ion at *m/z* 1314.841 and ion at 1328.856 can be seen in this figure as well as the high-resolution MS-MS spectra of *m/z* 1314.841 and *m/z* 1328.856.

Figs. S8 is a comparison of the high-resolution MS-MS of compounds **37 (***m/z* 1314.841) and **42** (*m/z* 1330.856).

Figs. S9 depicts the conserved core motif sequences of the condensation, adenylation and thiolation domains. Conserved sequences are highlighted.


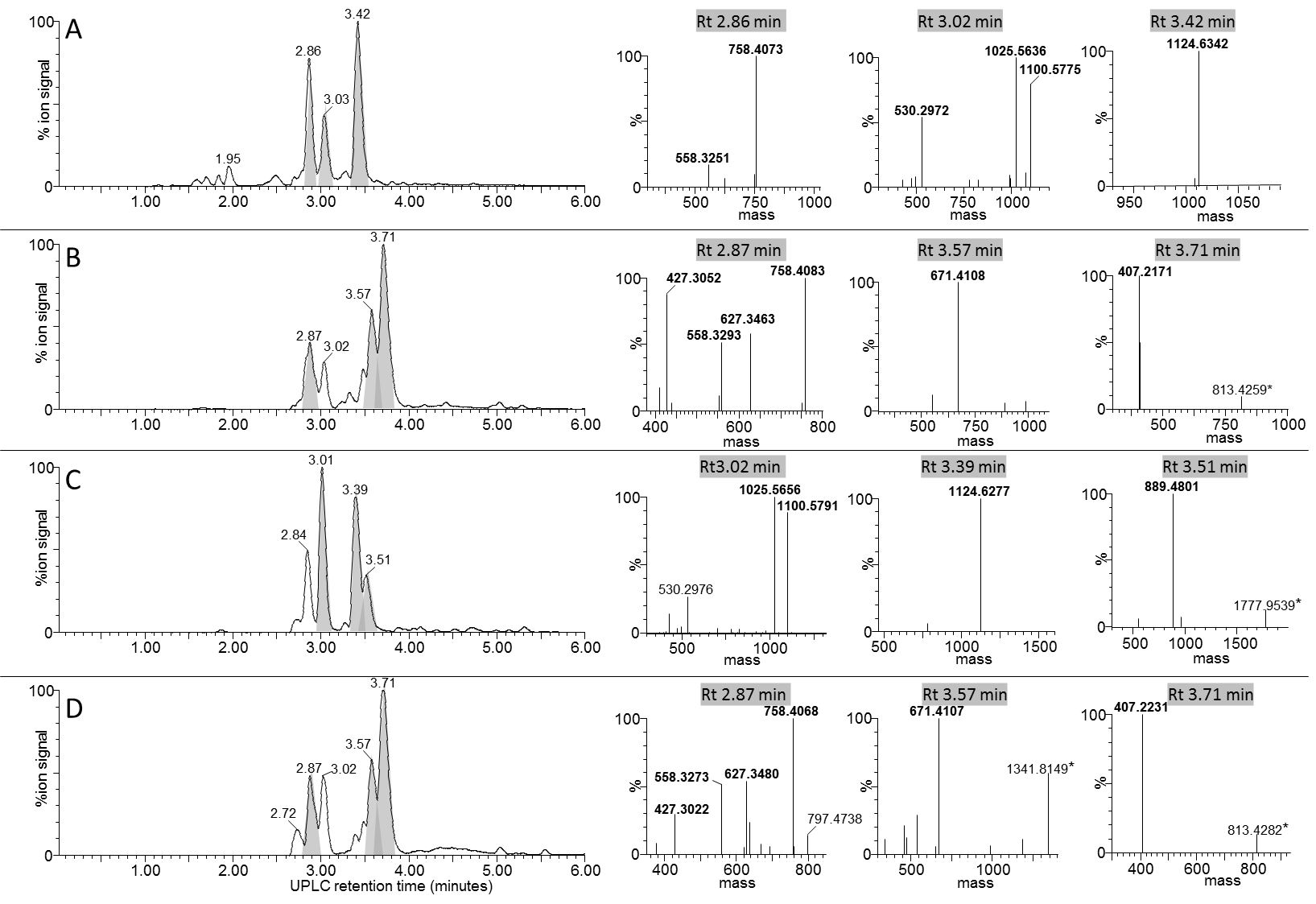


**FIGURE S1.** UPLC-MS of the early eluting components (0 to 6 min) from the four *X. khoisanae* J194 extracts that showed antibacterial activity: (A) NAA, (B) NAO, (C) AB (D) SM. The UPLC profiles are shown on left and the mass spectra from multi-protonated isotopic resolved spectra (deconvoluted with MassLynx 4.01 MaxEnt 3 algorithm) of the selected peaks shaded in grey are shown on right. The masses in the spectra are protonated masses of the most prominent detected compounds.


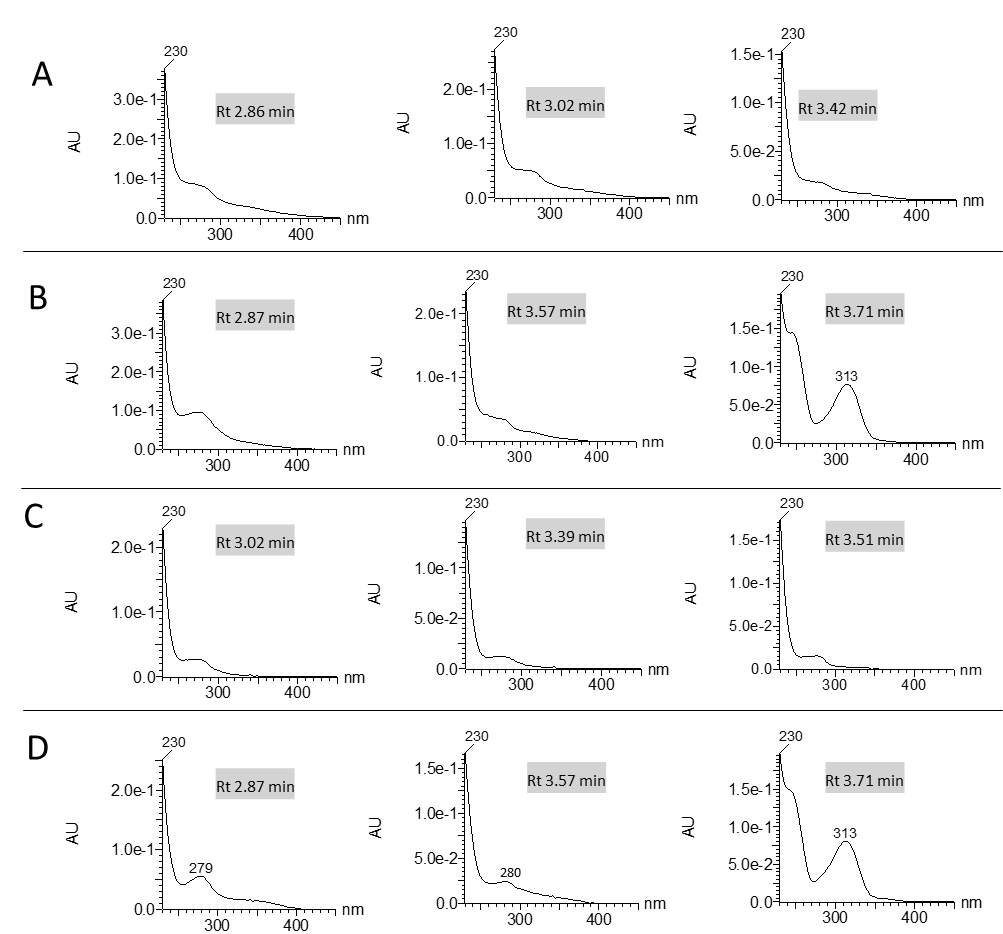


**FIGURE S2.** UV spectra of the early eluting components (0 to 6 min) from the four *X. khoisanae* J194 extracts that showed antibacterial activity: (A) NAA, (B) NAO, (C) AB (D) SM. The retention time correlate with the selected peaks selected in grey in Fig. S1.


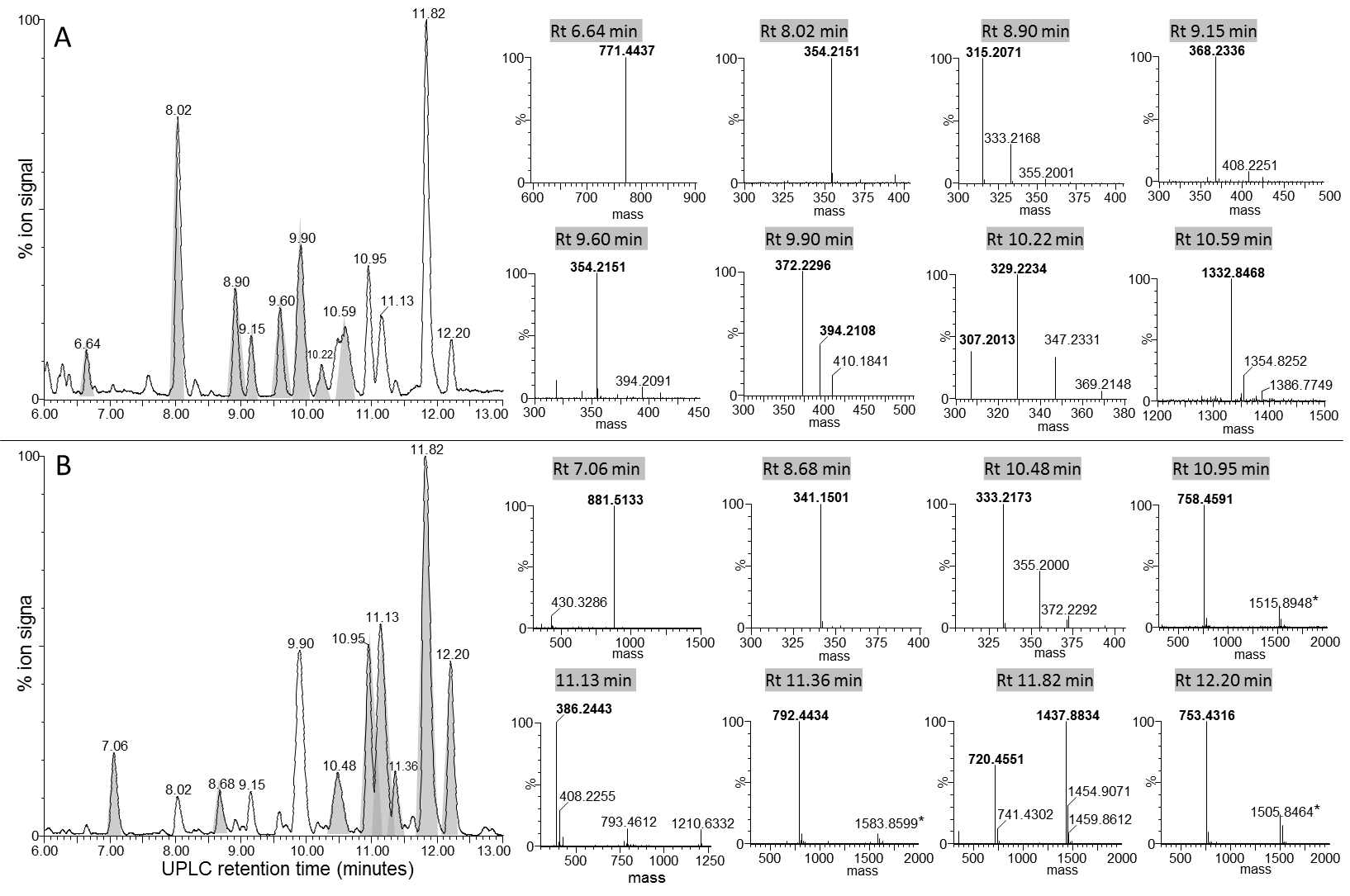


**FIGURE S3**. UPLC-MS of the mid-gradient eluting components (6 to 13 min) from the two *X. khoisanae* J194 extracts that showed antibacterial activity: (A) NAO and (B) SM. The UPLC profiles are shown on left and the extracted mass spectra from multi-protonated isotopic resolved spectra (deconvoluted with MassLynx 4.01 MaxEnt 3 algorithm) of the selected peaks shaded in grey are shown on the right. The masses in the spectra are protonated masses of the most prominent detected compounds.


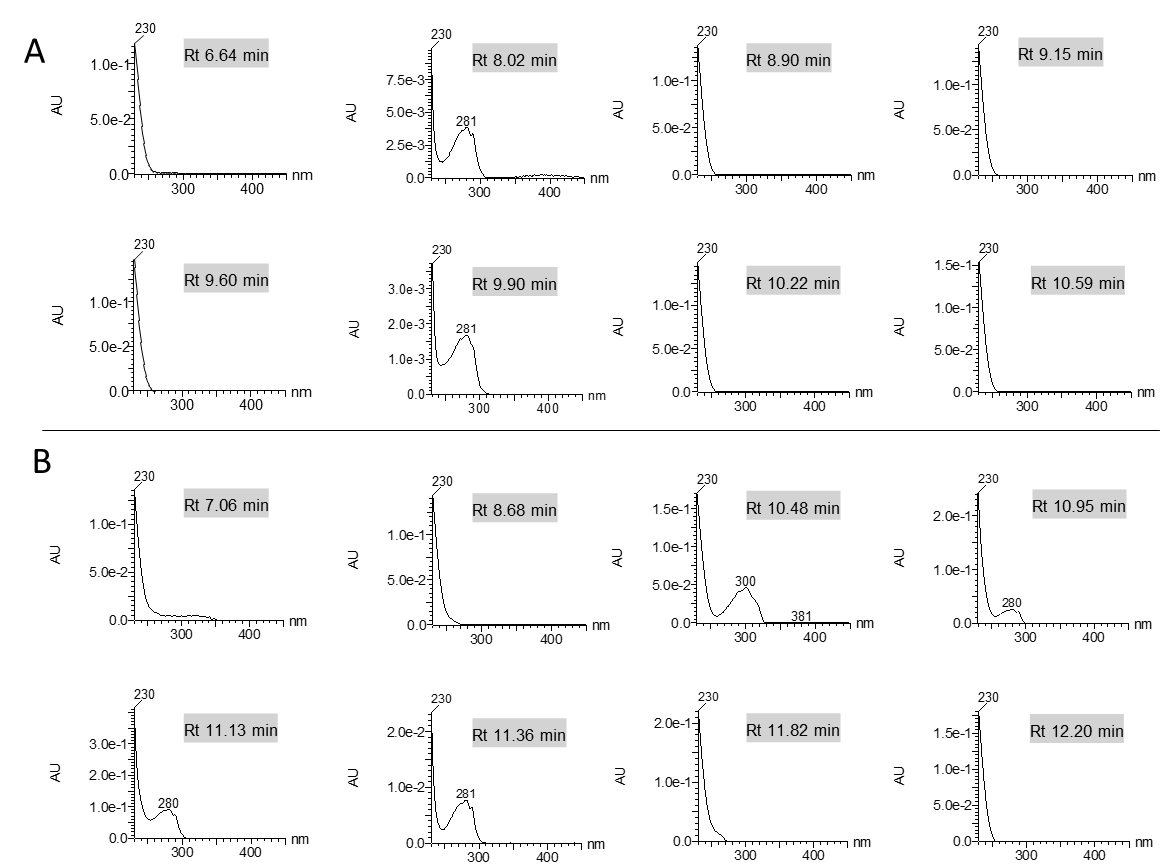


**FIGURE S4.** UV spectra of the early eluting components (6 to 13 min) from the two *X. khoisanae* J194 extracts that showed antibacterial activity: (A) NAO and (B) SM. The retention time correlate with the selected peaks selected in grey in Fig. S3.


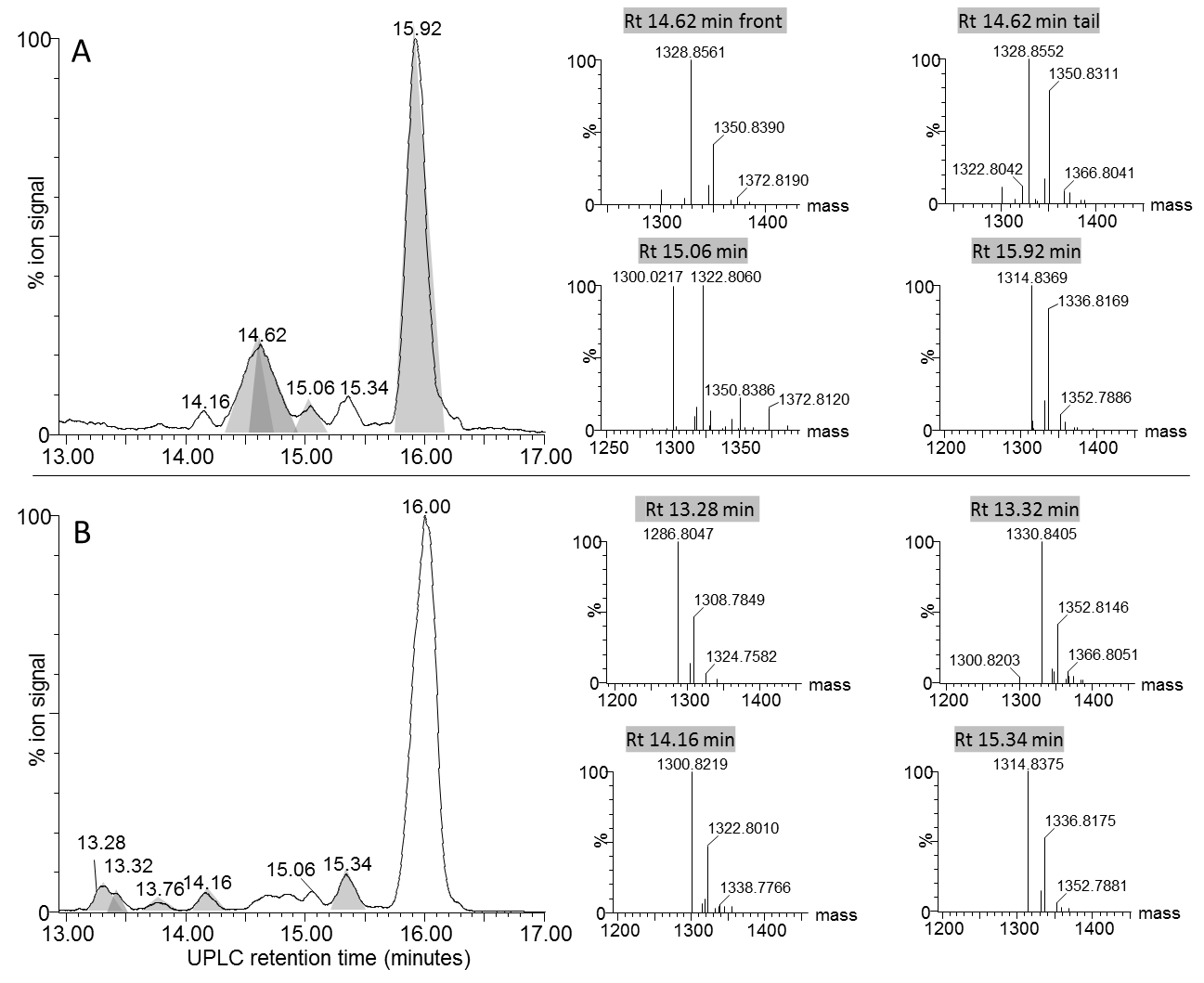


**FIGURE S5.** UPLC-MS of the late eluting components (13 to 17 min) from the *X. khoisanae* J194 extracts that showed antibacterial activity: (A) NAO (B) SM. The UPLC profiles are shown on the left and the extracted mass spectra from multi-protonated isotopic resolved spectra (deconvoluted with MassLynx 4.01 MaxEnt 3 algorithm) of the selected peaks are shaded in grey are shown on right. The masses in the spectra are protonated masses of the most prominent detected compounds.

**
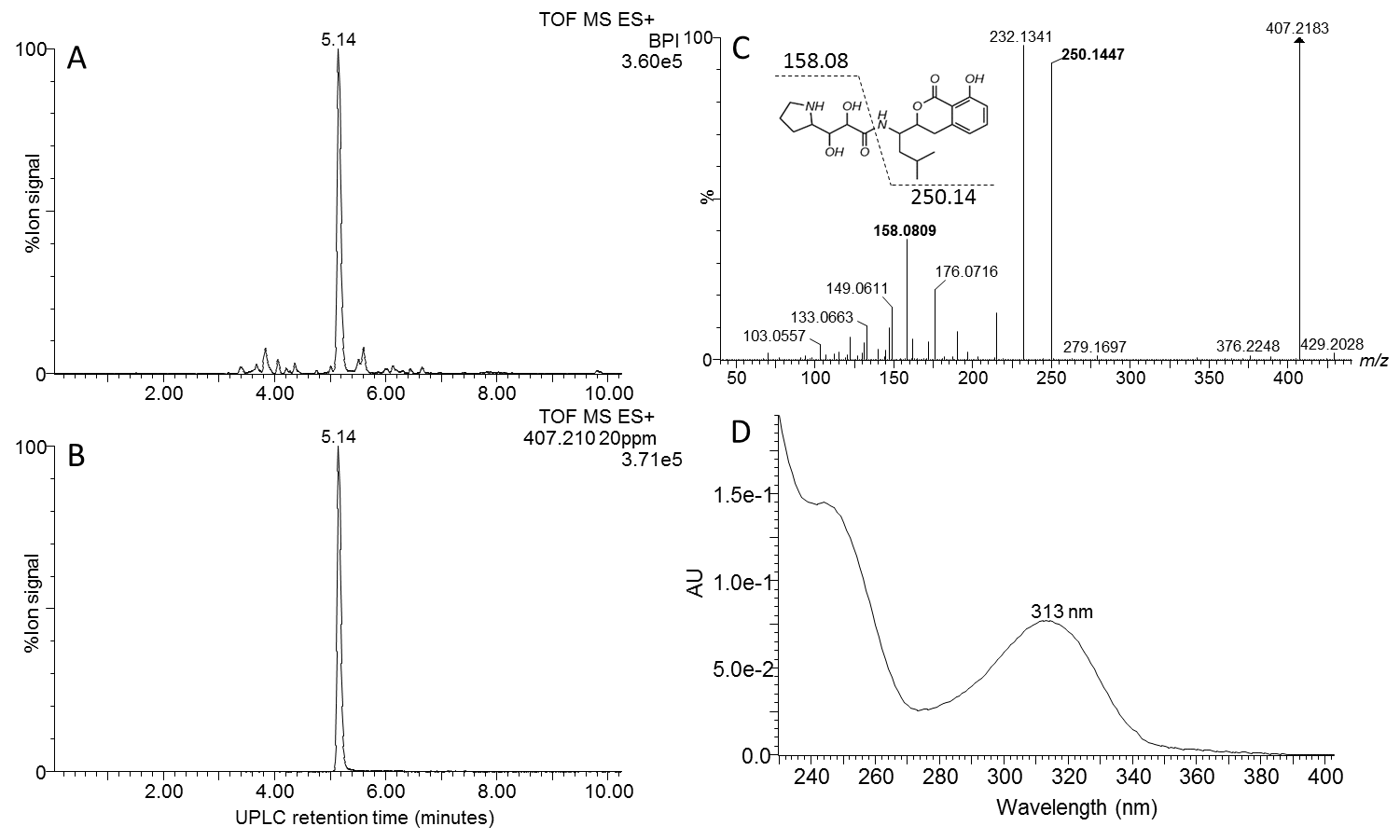
**

**FIGURE S6.** Structural analyses of compound **18** – xenocoumacin II. (A) UPLC-MS analysis of the purified compound, (B) the extracted *m/z* chromatogram of the xenocoumacin II ion with *m/z=* 407.210, (C) high resolution MS-MS spectrum of 407.210; (D) Uv spectrum of the xenocoumacin II.


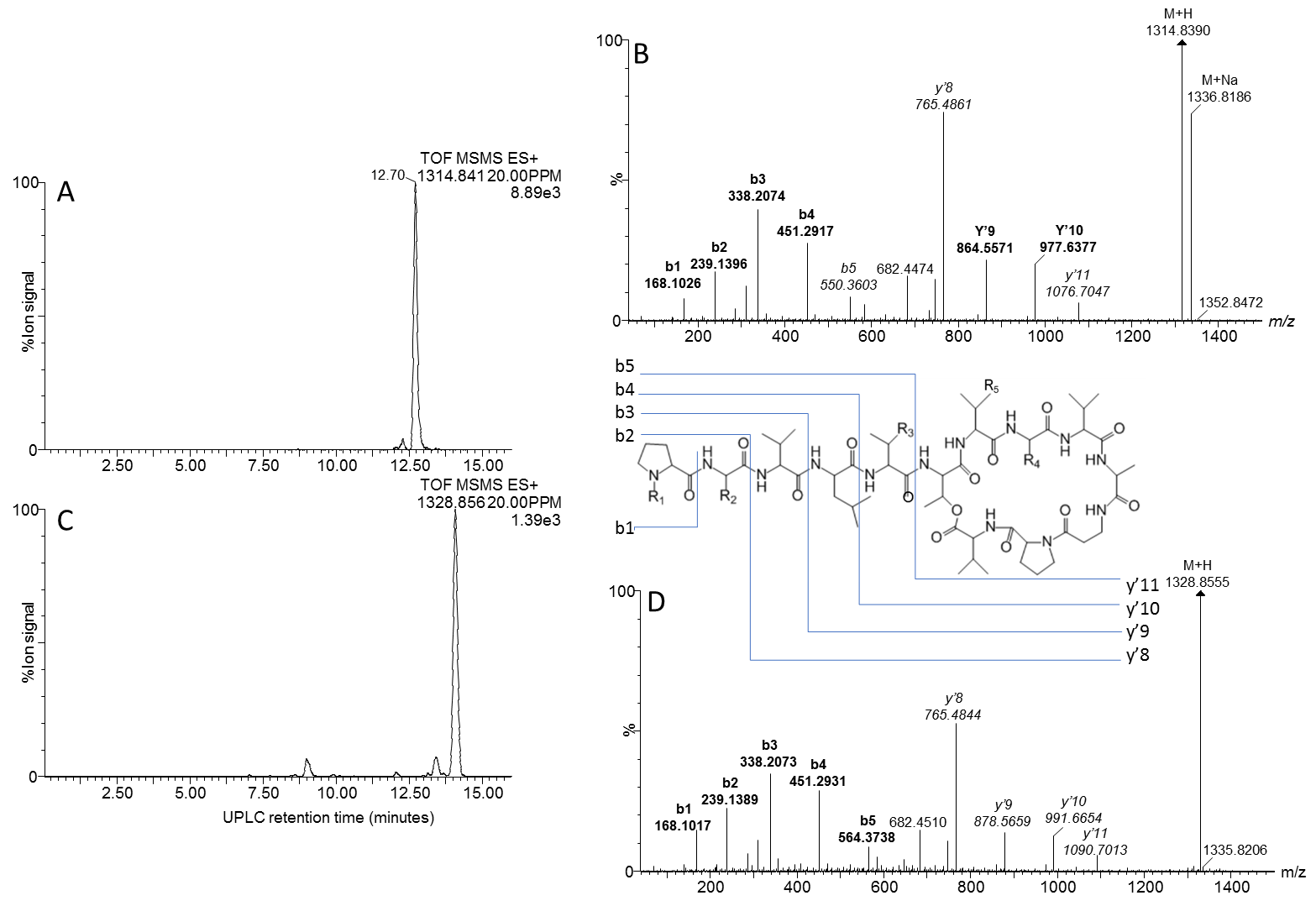


C

B

**FIGURE S7.** Structural analyses of compounds **38** and **42** – xenoamicins. UPLC-MS-MS analysis showing the m/z extracted chromatogram of ion at *m/z* 1314.841 (A) and ion at 1328.856 (B). High resolution MS-MS spectra of *m/z* 1314.841 (C) and *m/z* 1328.856 (D).


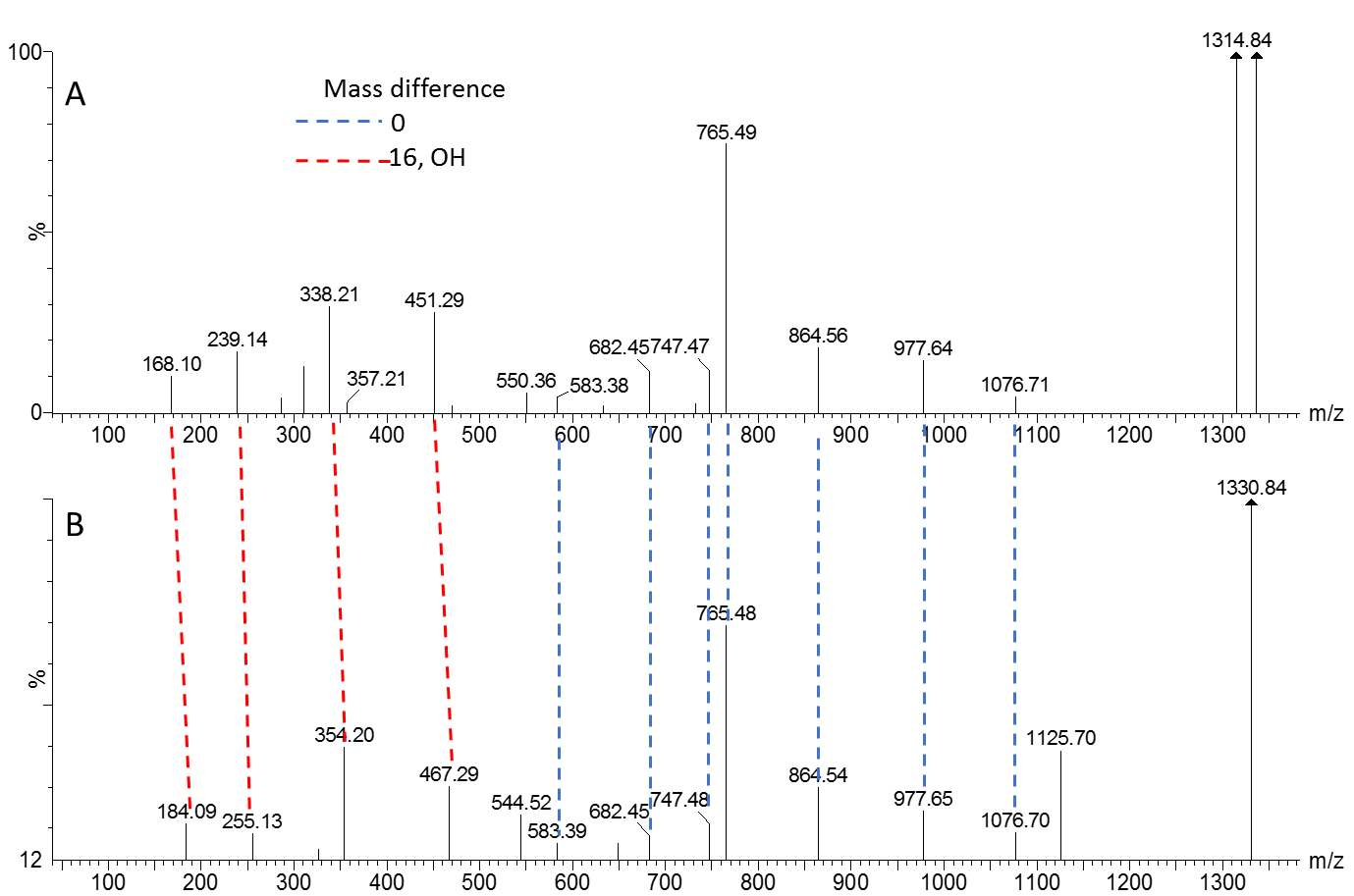


**FIGURE S8.** Comparison of the MS-MS of **37** and **42** – xenoamicins. High resolution MS-MS spectra of *m/z* 1314.841 (A) and *m/z* 1330.856 (B).

| C Domains | | | |
| --- | --- | --- | --- |
|  | Core/Motif 1 | Core/Motif 2 | Core/Motif 3 |
|  | SxAQxRxxxL | RHExLRTxF | MHHxISDGxS |
| SYNT 1 – MOD 1 | ------------------ | ------------------ | ------------------ |
| SYNT 1 – MOD 2 | HLAQPRDDAY | RFPILRTAF | QHHSIIDGWS |
| SYNT 2 – MOD 3 | ------------------ | ------------------ | ------------------ |
| SYNT 3 – MOD 4 | ------------------ | ------------------ | ------------------ |
| SYNT 3 – MOD 5 | SFAQQRLWFL | RQEILHTRF | HHHIISDGWS |
| SYNT 3 – MOD 6 | SFAQQRLWFL | RQEILRTRF | QHHIISDGWS |
| SYNT 4 – MOD 7 | ------------------ | ------------------ | ------------------ |
| SYNT 5 – MOD 8 | ------------------ | ------------------ | ------------------ |
| SYNT 5 – MOD 9 | SFAQQSLWFL | RHESLRTRF | LHHIITDGWS |
|  |  |  |  |
|  | Core/Motif 4 | Core/Motif 5 | Core/Motif 6 |
|  | YxDxAVW | XGxFVNTxxxR | xQDxPFE |
| SYNT 1 – MOD 1 | ------------------ | ------------------ | ------------------ |
| SYNT 1 – MOD 2 | AESEAYW | VGLYINTLPLM | HSLFVFE |
| SYNT 2 – MOD 3 | ------------------ | ------------------ | ------------------ |
| SYNT 3 – MOD 4 | ------------------ | ------------------ | ------------------ |
| SYNT 3 – MOD 5 | YADYAVW | MGFFVNTLALR | HQDLPFE |
| SYNT 3 – MOD 6 | YADYAVW | IGFFVNTLALR | HQDLPFE |
| SYNT 4 – MOD 7 | ------------------ | ------------------ | ------------------ |
| SYNT 5 – MOD 8 | ------------------ | ------------------ | ------------------ |
| SYNT 5 – MOD 9 | YADYAVW | MGFFVNTLALR | HQDLPFE |

| A Domains | | | | | |
| --- | --- | --- | --- | --- | --- |
|  | Core/Motif 1 | Core/Motif 2 | Core/Motif 3 | Core/Motif 4 | Core/Motif 5 |
|  | LxYxEL | LKAGxAYLxPxD | LAYxxYTSGxTGxPKG | FDxS | NxYGPTE |
| SYNT 1 – MOD 1 | LTYRQL | LKAGGAYVPIS | LAYIIYTSGTTGQPKG | FDAS | NLYGPTE |
| SYNT 1 – MOD 2 | LTYRQL | LKAGGAYVPIS | LAYIIYTSGTTGQPKG | FDGS | NAYGPTE |
| SYNT 2 – MOD 3 | LTYRQL | LKAGGAYVPIS | LAYIIYTSGTTGQPKG | FDAS | NQYGPTE |
| SYNT 3 – MOD 4 | LSYGEL | LKAGGAYVPLD | VAYVIYTSGSTGLPKG | FDVA | NHYGPTE |
| SYNT 3 – MOD 5 | LSYEEL | LKAGGAYVPLD | LAYVIYTSGSTGLPKG | FDAS | NAYGPTE |
| SYNT 3 – MOD 6 | LSYGEL | LKTGGAYVPLD | LAYVIYTSGSTGKPKG | FDFS | NMYGITE |
| SYNT 4 – MOD 7 | LSYDEL | LKAGGAYVPLD | RAYIIYTSGSTGLSKG | FDAA | HMYGPTE |
| SYNT 5 – MOD 8 | MSYGEL | LKAGGAYVPLD | LAYVIYTSGSTGLPKG | FDVA | NHYGPTE |
| SYNT 5 – MOD 9 | ISYGEL | LKAGGAYVPLD | LAYVIYTSGSTGQPKG | FDAS | NGYGPTE |
|  |  |  |  |  |  |
|  | Core/Motif 6 | Core/Motif 7 | Core/Motif 8 | Core/Motif 9 | Core/Motif 10 |
|  | GELxIxGxGxARGYL | YxTGDL | GRxDxQVKIRGxRIELGEIE | LPxYMxP | NGKxDR |
| SYNT 1 – MOD 1 | GELYIGGAGLARGYR | YKTGDL | GRNDFQVKIRGYRIELGEIE | LPEYMIP | NGKLDR |
| SYNT 1 – MOD 2 | GELYIGGAGLARGYL | YKTGDL | GRNDFQVKIRGYRIELGEIE | LPEYMLP | NGKLDR |
| SYNT 2 – MOD 3 | GELYIGGAGLARGYW | YKTGDR | GRNDFQVKIRGYRIELGEIE | LPEYMLP | NGKLNR |
| SYNT 3 – MOD 4 | GEIHIAGAGVARGYL | YKTGDL | GRNDFQVKLRGFRIELGEIE | LAEYMLP | NGKLDR |
| SYNT 3 – MOD 5 | GEIYIAGAGVARGYL | YRTGDL | GRNDFQVKLRGFRIELGEIE | LAEYMLP | NGKLDR |
| SYNT 3 – MOD 6 | GEIYIVGAGVTRGYL | YKTGDL | GRNDFQVKIRGFRIELGEIE | LAEYMLP | NGKLDR |
| SYNT 4 – MOD 7 | GEIYVAGAGVARGYL | YKTGDL | GRNDFQIKLRGFRIELGEIE | LAEYMLP | NGKTDR |
| SYNT 5 – MOD 8 | GEIHIAGAGVARGYL | YKTGDL | GRNDFQIKIRGFRIELGEIE | LAEYMLP | NGKLDR |
| SYNT 5 – MOD 9 | GEIHIAGAGVARGYL | YKTGDL | GRNDFQVKLRGFRIELGEIE | LAEYMIP | NGKLDR |

| Thiolation domain | |
| --- | --- |
|  | Core/Motif T |
|  | DxFFxLGGxSx |
| SYNT 1 – MOD 1 | DNFFRIGGDSI |
| SYNT 1 – MOD 2 | DNFFRIGGNSL |
| SYNT 2 – MOD 3 | DNFFRLGGNSL |
| SYNT 3 – MOD 4 | DHFFELGGHSL |
| SYNT 3 – MOD 5 | DHFFELGGHSL |
| SYNT 3 – MOD 6 | DHFFELGGHSL |
| SYNT 4 – MOD 7 | DHFFELGGHSL |
| SYNT 5 – MOD 8 | DHFFELGGHSL |
| SYNT 5 – MOD 9 | DHFFELGGHSL |

**FIGURE S9.** Alignment of conserved sequences of the (A) condensation domain, (B) adenylation domain and (C) thiolation domain (Marahiel et al., 1997).

**References**

Marahiel, M. A., Stachelhaus, T., and Mootz, H. D. (1997). Modular peptide synthetases involved in nonribosomal peptide synthesis. *Chem. Rev.* 97, 2651–2674. doi:10.1021/cr960029e.
